# *In vitro* evaluation of the protective effects of plant extracts against amyloid-beta peptide-induced toxicity in human neuroblastoma SH-SY5Y cells

**DOI:** 10.1371/journal.pone.0212089

**Published:** 2019-02-14

**Authors:** Ana Luiza Sereia, Marcelo Tempesta de Oliveira, Adrivanio Baranoski, Leila Larisa Medeiros Marques, Fabianne Martins Ribeiro, Raquel Garcia Isolani, Daniela Cristina de Medeiros, Danielly Chierrito, Danielle Lazarin-Bidóia, Acácio Antonio Ferreira Zielinski, Cláudio Roberto Novello, Celso Vataru Nakamura, Mário Sérgio Mantovani, João Carlos Palazzo de Mello

**Affiliations:** 1 Programa de Pós-Graduação em Ciências Farmacêuticas, Department of Pharmacy, Universidade Estadual de Maringá, Maringá, Paraná, Brazil; 2 Programa de Pós-Graduação em Genética e Biologia Molecular, Department of General Biology, Universidade Estadual de Londrina, Londrina, Paraná, Brazil; 3 Department of Chemical Engineering and Food Engineering, Universidade Federal de Santa Catarina, Florianópolis, Santa Catarina, Brazil; 4 Academic Department of Chemistry and Biology, Universidade Tecnológica Federal do Paraná, Francisco Beltrão, Paraná, Brazil; University of PECS Medical School, HUNGARY

## Abstract

Alzheimer’s disease (AD) is the most common form of dementia and has no cure. Therapeutic strategies focusing on the reduction of oxidative stress, modulation of amyloid-beta (Aβ) toxicity and inhibition of tau protein hyperphosphorylation are warranted to avoid the development and progression of AD. The aim of this study was to screen the crude extracts (CEs) and ethyl-acetate fractions (EAFs) of *Guazuma ulmifolia*, *Limonium brasiliense*, *Paullinia cupana*, *Poincianella pluviosa*, *Stryphnodendron adstringens* and *Trichilia catigua* using preliminary *in vitro* bioassays (acetylcholinesterase inhibition, antioxidant activity and total polyphenol content) to select extracts/fractions and assess their protective effects against Aβ_25–35_ toxicity in SH-SY5Y cells. The effect of the EAF of *S*. *adstringens* on mitochondrial membrane potential, lipid peroxidation, superoxide production and mRNA expression of 10 genes related to AD was also evaluated and the electropherogram fingerprints of EAFs were established by capillary electrophoresis. Chemometric tools were used to correlate the *in vitro* activities of the samples with their potential to be evaluated against AD and to divide extracts/fractions into four clusters. Pretreatment with the EAFs grouped in cluster 1 (*S*. *adstringens*, *P*. *pluviosa* and *L*. *brasiliense*) protected SH-SY5Y cells from Aβ_25-35_-induced toxicity. The EAF of *S*. *adstringens* at 15.62 μg/mL was able completely to inhibit the mitochondrial depolarization (69%), superoxide production (49%) and Aβ_25-35_-induced lipid peroxidation (35%). With respect to mRNA expression, the EAF of *S*. *adstringens* also prevented the *MAPT* mRNA overexpression (expression ratio of 2.387x) induced by Aβ_25–35_, which may be related to tau protein hyperphosphorylation. This is the first time that the neuroprotective effects of these fractions have been demonstrated and that the electropherogram fingerprints for the EAFs of *G*. *ulmifolia*, *L*. *brasiliense*, *P*. *cupana*, *P*. *pluviosa* and *S*. *adstringens* have been established. The study expands knowledge of the *in vitro* protective effects and quality control of the evaluated fractions.

## Introduction

Ongoing advances and developments in modern medicine and science have increased life expectancy, exponentially increasing the prevalence of senile disorders like dementia [1,2]. Worldwide, it is estimated that more than 47 million people are living with dementia. In 2018, the economic impact will be about US$3 trillion, becoming one of the biggest global public health and social problems [3].

Alzheimer’s disease (AD) is the most common form of dementia and is characterized by the progressive loss of memory, language skills and cognitive ability, ultimately leading to death [4–7]. Pathologically, AD is characterized by excessive deposition of extraneuronal amyloid-beta (Aβ) peptide plaques, intracellular neurofibrillary tangles, hyperphosphorylation of tau protein and neuronal cell death. Abnormal Aβ aggregation and the formation of reactive oxygen species (ROS) due to oxidative stress are the main factors responsible for the development and progression of AD [4–13].

Currently, the use of acetylcholinesterase (AChE) inhibitors is the main strategy used to alleviate the cognitive symptoms of AD [1,14–16]. Galantamine and rivastigmine are examples of two licensed anti-AD drugs based on plant-derived natural products. However, these treatments do not halt or delay disease progression [1,5,14–16], which justifies the search for therapeutic agents acting at other pathologic levels [1,2,17].

Compounds that are able to reduce excessive levels of ROS, Aβ production, oligomerization, neurotoxicity and inflammation induced by Aβ and inhibit tau protein hyperphosphorylation are potential targets for the discovery of new drugs with anti-AD properties [1,2,6,12,18]. A number of medicinal plants in the form of extracts, fractions, or isolated compounds have been reported as presenting possible anti-AD activity in preclinical and clinical studies [5]. Several authors have suggested that some types of alkaloids, saponins, glucosides, terpenoids, phenolic acids, flavonoids and extracts with antioxidant activity have protective effects against Aβ-induced toxicity in cell and animal models [1,2,4–7,11,12,18,19].

Among the Brazilian herbal species studied by our research group, *Guazuma ulmifolia* Lam. (Mutamba), *Limonium brasiliense* (Boiss.) Kuntze (Baicuru), *Paullinia cupana* Kunth (Guaraná), *Poincianella pluviosa* (DC.) L.P. Queiroz (Sibipiruna), *Stryphnodendron adstringens* (Mart.) Coville (Barbatimão) and *Trichilia catigua* A. Juss. (Catuaba) stand out due to their chemical profiles and biological potential [20–33]. However, to date, the neuroprotective effects of these species against Aβ toxicity have not been evaluated *in vitro* or *in vivo*. Thus, the aim of the present study was to evaluate the selected crude extracts (CEs) and their respective ethyl-acetate fractions (EAFs) using different preliminary *in vitro* bioassays related to AD and to apply chemometric tools to selected extracts/fractions to assess their protective effect against Aβ peptide toxicity in human neuroblastoma SH-SY5Y cells. The effect of the EAF of *S*. *adstringens* on mitochondrial membrane potential, lipid peroxidation, ROS production and mRNA expression of 10 genes related to AD was also evaluated. In addition, the chemical fingerprinting of EAFs was carried out by capillary electrophoresis to contribute to quality control of the evaluated plant fractions.

## Results and discussion

### Antioxidant activity, total polyphenol content and AChE inhibitory activity

The investigation of plant extracts, fractions or derived substances usually starts with carefully selected screening *in vitro*, followed by relevant *in vivo* models, with the aim of validation and reducing the number of animal tests [34]. We evaluated six selected CEs and their respective EAFs for antioxidant activity, total polyphenol content (TPC) and AChE inhibitory activity. The data from all tests are compiled in Table 1.

**Table 1:** *In vitro* antioxidant assays, total polyphenol content (TPC), and acetylcholinesterase (AChE) inhibitory activity in selected plant extracts and their semipurified fractions.

		Antioxidant assays					
	Plant Species	DPPH IC_50_ (μg/mL)	Xanthine oxidase system IC_50_ (μg/mL)	FRAP (mmol Trolox/ g sample)	ABTS (mmol Trolox/ g sample)	TPC (mg GAE/ g sample)	AChE inhibitory activity IC_50_ (mg/mL)
Crude extract	*Guazuma ulmifolia*	8.85^*e*^ ± 0.27	1.29^b^ ± 0.03	5.27^*gh*^ ± 0.18	2.99^*ef*^ ± 0.12	469.34^*ef*^ ± 17.32	2.27^b^ ± 0.08
	*Limonium brasiliense*	14.86^a^ ± 0.19	0.18^*h*^ ± 0.01	5.53^*fg*^ ± 0.20	2.78^*g*^ ± 0.01	329.67^*i*^ ± 1.79	1.56^*d*^ ± 0.08
	*Paullinia cupana*	7.84^*f*^ ± 0.31	1.11^*c*^ ± 0.05	4.43^*j*^ ± 0.09	2.23^*i*^ ± 0.10	453.34^*fg*^ ± 9.09	3.38^a^ ± 0.14
	*Poincianella pluviosa*	11.09^*c*^ ± 0.41	1.23^b^ ± 0.07	4.78^*ij*^ ± 0.11	2.08^*j*^ ± 0.08	369.07^*h*^ ± 5.89	0.25^*i*^ ± 0.01
	*Stryphnodendron adstringens*	9.65^*d*^ ± 0.47	1.70^a^ ± 0.03	5.79^*ef*^ ± 0.08	3.27^*d*^ ± 0.06	488.06^*e*^ ± 15.35	0.58^*g*^ ± 0.00
	*Trichilia catigua*	5.51^*h*^ ± 0.27	0.86^*d*^ ± 0.05	5.51^*fg*^ ± 0.17	3.14^*d*^ ± 0.07	543.85^*c*^ ± 10.56	2.14^*c*^ ± 0.06
Ethyl-acetate fraction	*Guazuma ulmifolia*	10.10^*d*^ ± 0.48	0.75^*e*^ ± 0.03	6.41^*d*^ ± 0.25	3.12^*de*^ ± 0.08	552.43^*c*^ ± 12.22	1.14^*e*^ ± 0.02
	*Limonium brasiliense*	5.41^*h*^ ± 0.08	0.69^*e*^ ± 0.02	6.18^*de*^ ± 0.23	6.64^a^ ± 0.11	551.26^*c*^ ± 3.10	0.47^*h*^ ± 0.01
	*Paullinia cupana*	8.04^*f*^ ± 0.25	0.50^*f*^ ± 0.03	5.08^*hi*^ ± 0.16	2.47^*h*^ ± 0.04	578.18^b^ ± 17.24	1.19^*e*^ ± 0.04
	*Poincianella pluviosa*	6.18^*g*^ ± 0.11	0.30^*g*^ ± 0.01	11.49^b^ ± 0.22	4.05^b^ ± 0.13	523.56^*d*^ ± 4.43	0.61^*g*^ ± 0.00
	*Stryphnodendron adstringens*	12.46^b^ ± 0.59	0.30^*g*^ ± 0.01	13.66^a^ ± 0.38	3.70^*c*^ ± 0.11	436.17^*g*^ ± 4.73	0.62^*g*^ ± 0.03
	*Trichilia catigua*	5.29^*h*^ ± 0.09	0.48^*f*^ ± 0.02	9.37^*c*^ ± 0.45	2.92^*fg*^ ± 0.03	735.80^a^ ±18.74	0.77^*f*^ ± 0.04
p-value		< 0.001	< 0.001	< 0.001	< 0.001	< 0.001	< 0.001

Note: Results expressed as mean ± SD (n = 3). Probability values obtained by one-way analysis of variance (ANOVA).

^ab^ Different superscript letters in the same column represent statistically different results (p < 0.05).

Given that elevated ROS levels are associated with oxidative damage, increased Aβ deposition, the formation of senile plaques and cell death [14], the antioxidant activity of the extracts was evaluated. Four different antioxidant test models were estimated: the 2,2'-diphenyl-1-pocrylhydrazyl (DPPH) radical scavenging assay, the ferric reducing antioxidant power (FRAP) assay, the 2,2'-azino-bis(3-ethylbenzothiazoline-6-sulphonic acid) (ABTS) antioxidant assay and the xanthine oxidase activity assay. Alam and colleagues [35] recommend employing different antioxidant test models as they vary in several respects.

The results showed that the inhibition of DPPH and the xanthine oxidase system ranged from 5.29 to 14.86 and 0.18 to 1.70 μg/mL, respectively. The FRAP and ABTS results ranged from 5.08 to 13.66 and 2.08 to 6.64 mmol Trolox/g sample, respectively. Due to the differences between the tests, it is difficult to compare one method directly with another [35] and consequently to state which extract or EAF presents the highest antioxidant activity. In general, we can say that most fractions obtained presented better antioxidant activity compared to the CEs of origin. With respect to TPC, the results ranged from 329.67 to 735.80 mg of gallic acid equivalent [GAE]/g sample. As in the case of antioxidant activity, most EAF samples showed higher polyphenol content compared to their respective CEs. Indeed, many studies have shown a significant correlation between antioxidant capacity and TPC [19,21,36,37], reinforcing our results.

The CEs and EAFs were also screened for AChE inhibition. The IC_50_ ranged from 0.25 to 3.38 mg/mL. With the exception of the species *P*. *pluviosa* and *S*. *adstringens*, the EAFs also presented better inhibitory activity of AChE compared to the CEs of origin, as shown in other studies [16]. Some authors [38–40] have suggested a synergic correlation between the presence of polyphenols and antioxidant and anti-AChE activities *in vitro*, as we observed. The comparison of the quantitative results of these *in vitro* bioassays with the available literature is difficult, owing to the distinct nature of the extracting solvents and procedures [40]. Morais et al. [38] found that ethanol extract from *G*. *ulmifolia* leaves exhibit significant anti-AChE activity and suggested that this result can be attributed to the presence of flavonoids and phenolic acids with recognized antioxidant potential in the extract. Rodrigues et al. [39] evaluated an another *Limonium* specie and showed that the infusions and decoctions of *L*. *algarvense* flowers powder had high phenolic contents and strong antioxidant activity and capacity to inhibit AChE (IC_50_ values of 0.22 to 0.39 mg/mL). Bernardo et al. [40] evaluated the AChE inhibition of an aqueous extract of *T*. *catigua* in rat brain tissue homogenates and found that the extract, which is rich in flavanols and derivatives, demonstrated an inhibitory effect over the enzymatic activity, besides a valuable antioxidant activity. Ruchel et al. [41] evaluated AChE activity in different brain structures of rats treated with *P*. *cupana* seed powder and suggested that guaraná powder may be a source of phytochemicals that can be used as an adjuvant therapy in the management of cognitive disorders. Recently, a study showed that the protective effect of *P*. *cupana* hydroalcoholic extract in *Caenorhabditis elegans* models of AD is associated with antioxidant activity and the considerable number of polyphenol constituents [42]. This study is the first to evaluate AChE inhibitory activity using extracts/fractions of *L*. *brasiliense*, *P*. *pluviosa*, *S*. *adstringens* and the bark of *G*. *ulmifolia*.

### Use of chemometric tools to select extracts/fractions for investigation

To correlate the *in vitro* activities of the samples under study with their potential for investigation in future studies related to AD, principal component analysis (PCA) was applied to examine their distribution. Several authors have used multivariate statistical tools to rationalize the choice of potential extracts/fractions for investigation and have shown that this method is a suitable approach to check for similarities and differences and to create natural groupings among samples [22,30,37].

The first principal component (PC1) accounted for up to 37.75% of the total variance, while PC2 accounted for 28.04%, totalling 65.79% of the total variance (S1 Fig). PC1 separated the samples according to differences observed in the DPPH (0.536), ABTS (-0.681), FRAP (-0.688), xanthine oxidase system (0.533), TPC (-0.669) and AChE inhibitory activity (0.557) assays. PC2 distinguished samples according to differences observed in the levels of DPPH (-0.804) and TPC (0.596). Through assessment of the scatter plots (*Scores* and *Loadings*, S1 Fig), it was possible to distinguish which plant extracts were most strongly associated with phenolic compounds, antioxidant activity and AChE inhibitory activity. The samples obtained as EAFs showed the highest content of phenolic compounds and better antioxidant and AChE inhibitory activity, as expected and already discussed [21,36,37]. The EAFs also showed better AChE inhibitory activity, probably because the relative abundance of the active constituents in these fractions was higher [34].

In addition, hierarchical cluster analysis (HCA) was used to evaluate the similarity/dissimilarity of the samples; four clusters were suggested. The means for each response variable were also compared between the clusters (Table 2). First, separation between EAFs and CEs was observed (clusters 1 and 2 *versus* clusters 3 and 4), confirming the PCA results. In the EAF, the plants comprising cluster 1 were *S*. *adstringens*, *P*. *pluviosa* and *L*. *brasiliense* presented the best results regarding AChE inhibitory activity (0.57 ± 0.09 mg/mL). In cluster 2, the fractions from *G*. *ulmifolia*, *P*. *cupana* and *T*. *catigua* had the highest TPC (622.14 ± 99.27 mg GAE/g sample), although there was no statistically significant difference between clusters 1 and 2. Regarding antioxidant activity, there was no statistically significant difference between the four clusters (p > 0.05). Ali and colleagues [14] suggest that plant extracts with potent free radical scavenging properties (IC_50_ < 10 μg/mL in the DPPH assay) are able to reduce oxidative stress and may be evaluated against AD or other ageing-related diseases. Zeng et al. [12] showed that icariin, a flavonoid from *Epimedium brevicornu* Maxim., significantly reduced Aβ_25-35_-induced cytotoxicity and apoptosis in PC12 cells. Yu et al. [18] demonstrated that a water extract of *Salvia miltiorrhiza* Bunge, which is rich in diterpenoids and phenolic acids, provides substantial neuroprotection against Aβ_25-35_-induced neurotoxicity in SH-SY5Y cells, at least in part by inhibiting oxidative stress and may have potential effects in preventing or relieving AD.

**Table 2:** Mean values of antioxidant activity, total polyphenol content (TPC) and acetylcholinesterase (AChE) inhibitory activity from crude extracts and semipurified fractions classified using hierarchical cluster analysis.

Analysis	Cluster 1 EAFs of *S*. *adstringens*, *P*. *pluviosa*, and *L*. *brasiliense*	Cluster 2 EAFs of *G*. *ulmifolia*, *P*. *cupana*, and *T*. *catigua*	Cluster 3 CEs of *S*. *adstringens*, *P*. *pluviosa*, and *L*. *brasiliense*	Cluster 4 CEs of *G*. *ulmifolia*, *P*. *cupana*, and *T*. *catigua*	p-value*	p-value**
**DPPH** IC_50_ (μg/mL)	8.02 ± 3.87	7.81 ± 2.41	11.87 ± 2.69	7.40 ± 1.71	0.37	0.25
**Xanthine oxidase system** IC_50_ (μg/mL)	0.43 ± 0.23	0.57 ± 0.15	1.04 ± 0.78	1.09 ± 0.21	0.05	0.23
**FRAP** (mmol Trolox/g sample)	10.44 ± 3.84	6.95 ± 2.20	5.37 ± 0.52	5.07 ± 0.57	0.04	0.09
**ABTS** (mmol Trolox/g sample)	4.79 ± 1.61	2.84 ± 0.33	2.71 ± 0.59	2.79 ± 0.49	0.03	0.10
**TPC** (mg GAE/g sample)	503.67^ab^ ± 60.07	622.14^a^ ± 99.27	395.60^b^ ± 82.46	488.84^ab^ ± 48.31	0.39	0.04
**AChE inhibitory activity** IC_50_ (mg/mL)	0.57^*c*^ ± 0.09	1.04^b^ ± 0.23	0.79^b^^*c*^ ± 0.67	2.60^a^ ± 0.68	0.05	< 0.01

Note: Results expressed as mean ± standard deviation (SD).

* Probability values obtained from Levene’s test of homogeneity of variances.

** Probability values obtained from one-way ANOVA or the Kruskal-Wallis test.

^ab^ Different superscript letters in the same line represent statistically different results (p < 0.05).

Several authors have also reported that fractions that have the potential to inhibit AChE and are rich in polyphenols might have neuroprotective effects and thus may be used to prevent or postpone the onset of degenerative diseases such as AD [1,2,5,35,39]. This suggests that the EAFs in clusters 1 and 2 should preferably be used in further studies to explore their potential for the treatment of AD since they present better AChE inhibitory activity and higher polyphenol content.

Among the CEs, clusters 3 and 4 were grouped similarly to the EAF groups. Cluster 3 showed a better result for AChE inhibitory activity (0.79 ± 0.67 mg/mL). Regarding TPC, no statistically significant difference was observed between the two groups (p > 0.05). However, cluster 4 showed a slightly larger mean (488.84 ± 48.31 mg GAE/g sample). It is possible that a non-linear correlation [19] or an undetermined number of secondary metabolites besides polyphenols with different mechanisms of action may be involved in the biological activities of the extracts and semipurified fractions [14], which may explain this finding.

### Neuroprotective effects of the EAFs of *L*. *brasiliense*, *P*. *pluviosa* and *S*. *adstringens*

In this work, we aimed to evaluate the neuroprotective effects of the species grouped in cluster 1. We employed human neuron-like SH-SY5Y cells as an *in vitro* model to assess the protective effects of the EAFs of *L*. *brasiliense*, *P*. *pluviosa* and *S*. *adstringens* in a cellular system. To the best of our knowledge, this is the first study to evaluate the neuroprotective effects of these fractions. Aβ has been widely adopted as an inducer of neuronal injury to analyse the protective potential and mechanisms of new pharmacotherapies [18]. The Aβ_25–35_ peptide is the most neurotoxic fragment derived from full-length Aβ_1–42_ and mimics many of the oxidative properties of the native peptide; thus, it is often used for the *in vitro* study of various drugs predicted to modulate Aβ toxicity [10,12,18,43]. This peptide displays rapid aggregation properties, forms stable fibrils and is neurotoxic immediately upon dissolution [10].

First, we investigated the effects of the EAFs on cell viability in the range of 7.81 to 1.0 x 10^3^ μg/mL (S2 Fig). The respective fractions at concentrations of 7.81 to 62.5 μg/mL alone did not cause any apparent cytotoxicity after 24 h of treatment, measured by the 3-(4,5-dimethylthiazol-2-yl)-2,5-diphenyltetrazolium bromide (MTT) assay compared to that of the vehicle-treated control. The EAFs of *P*. *pluviosa*, *L*. *brasiliense* and *S*. *adstringens*, at concentrations of 125, 500 and 1.0 x 10^3^ μg/mL, respectively, were cytotoxic to SH-SY5Y cells. The comparison of these results with the available literature is also difficult, once it is not possible to directly extrapolate or correlate results from distinct species, mainly due to the distinct extracts composition. For example, an aqueous extract of *T*. *catigua* reduced the metabolic activity of SH-SY5Y cells at concentrations above 500 μg/mL [40], while a hydroethanolic extract from *Euterpe oleracea* caused changes in SH-SY5Y morphology at a concentration of 50 μg/mL [19]. Thus, we evaluated the EAFs separately and chose common non-cytotoxic concentrations of EAFs (7.81 to 31.25 μg/mL) to perform this assay.

**Fig 1:**
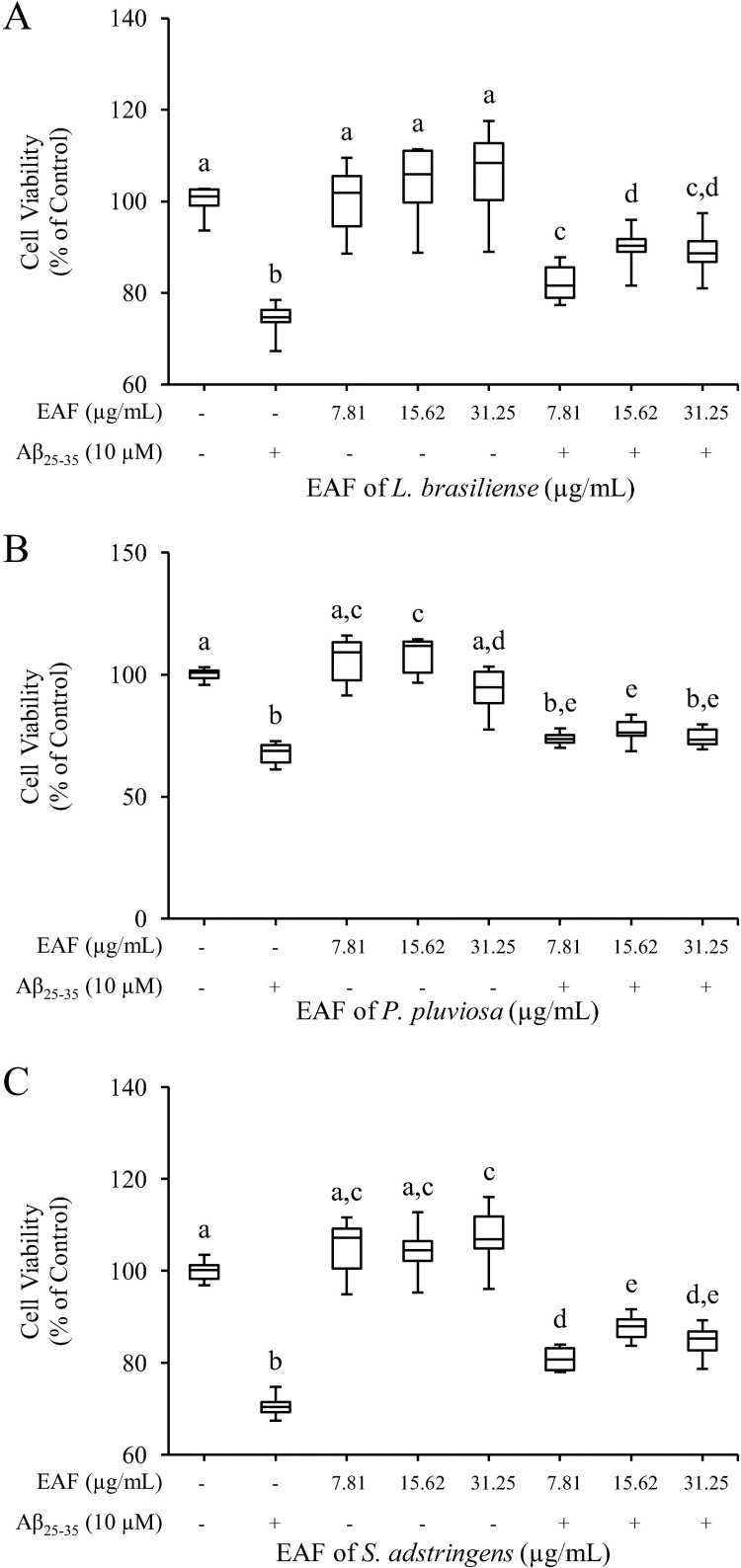
Effects of the EAFs of *Limonium brasiliense*, *Poincianella pluviosa* and *Stryphnodendron adstringens* on Aβ_25-35_-induced cytotoxicity. SH-SY5Y neuroblastoma cells were pretreated with different concentrations (7.81, 15.62 and 31.25 μg/mL) of EAF of *L*. *brasiliense* (A), EAF of *P*. *pluviosa* (B) and EAF of *S*. *adstringens* (C) for 2 h, followed by subsequent treatment with 10 μM Aβ_25-35_- for 24 h. Cell viability was measured by MTT reduction assay. Data are presented as mean ± SD of six replicates. ^ab^ Different superscript letters on the top of the bars indicates statistically significant differences between the multiple conditions comparison by ANOVA followed by Tukey’s test (p < 0.05), conducted in GraphPad Prism 5 software.

SH-SY5Y cells were pretreated with/without the non-cytotoxic concentrations of EAFs (7.81 to 31.25 μg/mL) for 2 h and incubated with 10 μM Aβ_25–35_ for 24 h. As shown in Fig 1, Aβ_25–35_ reduced cell viability by about 70%, which is consistent with other studies [9,11]. However, we observed that pretreatment with all the evaluated concentrations of EAFs of *L*. *brasiliense* and *S*. *adstringens* were able to attenuate the loss of cell viability compared with the Aβ_25–35_ group. Increased concentrations of these EAFs, from 7.81 to 15.62 μg/mL, exerted an additive protective effect (p < 0.05), indicating a dose-dependent action that was not further observed after an increase in the concentration of both fractions to 31.25 μg/mL. The EAF of *P*. *pluviosa* protected SH-SY5Y cells from Aβ-induced cytotoxicity only at a concentration of 15.62 μg/mL. However, the mechanisms by which these EAFs mediated their therapeutic effects against Aβ_25–35_ *in vitro* were unclear.

Among the evaluated fractions, we chose treatment with 15.62 μg/mL of the EAF of *S*. *adstringens* for the following studies.

### Effects of the EAF of *S*. *adstringens* on mitochondrial membrane potential, ROS production and lipid peroxidation

It is known that exposure of SH-SY5Y cells to Aβ_25–35_ may induce tau protein hyperphosphorylation and contribute to superoxide production, which can result in a free radical attack on membrane phospholipids, leading to a loss of mitochondrial membrane potential, modified proteins, damaged DNA and severe apoptosis [8,9,11–13,18]. Elevated ROS levels are also associated with oxidative damage, increased deposition of Aβ, the formation of senile plaques and cell death [14]. Neuronal cells are particularly susceptible to the actions of ROS and nitrogen species due to their high metabolic activity, low antioxidant capacity and non-replicative nature [19].

Dey et al. [5] showed that a number of herbal extracts, fractions, phytochemicals and herbal formulations may possess anti-AD properties via their antioxidant, anti-inflammatory, anti-apoptotic and anti-acetylcholinesterase activities, resulting in a reduction in Aβ-induced toxicity. In animal AD models, they may also increase learning and memory and prevent dementia [1,5].

As previously shown, the EAFs of *L*. *brasiliense*, *P*. *pluviosa* and *S*. *adstringens* exhibit anti-AChE activity and considerable polyphenol content, as well as antioxidant activity, suggesting that these EAFs may protect neuroblastoma cells against Aβ-induced oxidative damage, at least in part, by increasing the cellular redox potential.

**Fig 2:**
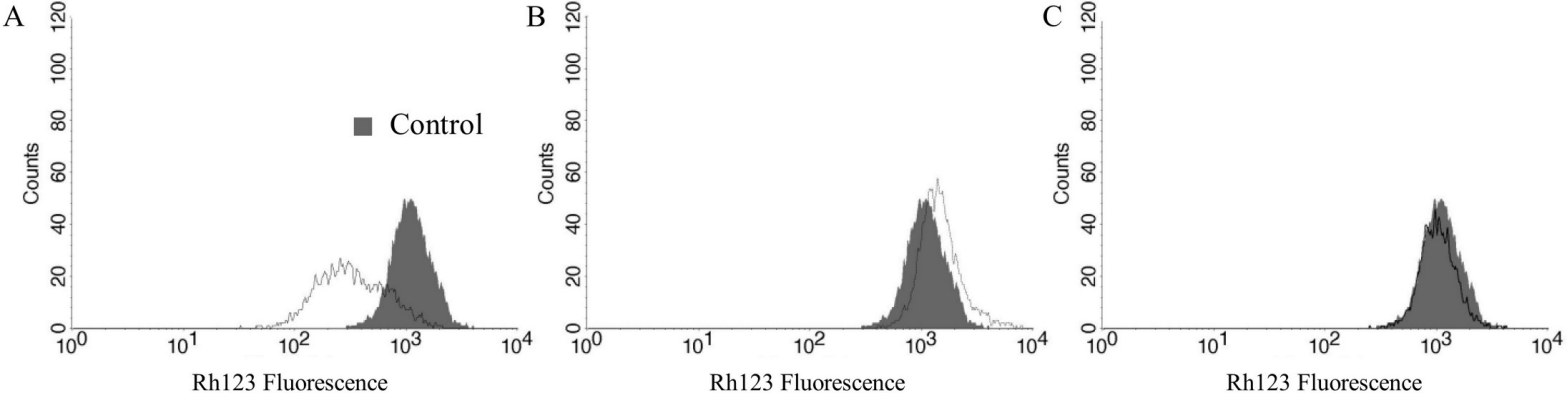
Effects of the EAF of *Stryphnodendron adstringens* on Aβ_25-35_-induced mitochondrial membrane depolarization in SH-SY5Y cells. Mitochondrial membrane potential was evaluated in treated SH-SY5Y cells using rhodamine 123 (Rh 123) staining, which accumulates within mitochondria. (A) 10 μM Aβ_25–35_ impact control. (B) EAF of *S*. *adstringens* control at 15.62 μg/mL. (C) Pretreatment with EAF of *S*. *adstringens* at 15.62 μg/mL for 2 h followed by treatment with 10 μM Aβ_25–35_ for 24 h. Control group (untreated cells) is also shown. Flow cytometric histograms for Rh123 fluorescence from at least three independent experiments are depicted.

Consistently, the mitochondrial membrane potential (ΔΨm) assay showed that the pretreatment with EAF of *S*. *adstringens* at 15.62 μg/mL protected cells against Aβ-induced mitochondrial depolarization. As shown in Fig 2 and Table 3, Aβ_25–35_ induced a significant (69%) decrease in total rhodamine 123 (Rh123) fluorescence intensity in the SH-SY5Y cells compared with the untreated control (p < 0.05), indicating mitochondrial depolarization, as previously reported by other authors [8,13]. The EAF of *S*. *adstringens* alone did not induce changes in ΔΨm compared with the control group and pretreatment for 2 h was able completely to inhibit Aβ-induced mitochondrial membrane depolarization (p < 0.05).

**Table 3:** Mitochondrial membrane potential assay in treated SH-SY5Y cells stained with Rh123.

	Median	Index of variation (IV^a^)
Control	991.05	0.00
Aβ_25–35_ impact control	305.05*	-0.69
EAF of *S*. *adstringens* control	1,084.32	0.09
Aβ_25–35_ + EAF of *S*. *adstringens*	950.67	-0.04

IV^a^ = (M_T_—M_C_)/M_C_, where M_T_ corresponds to the median of the fluorescence for treated cells and M_C_ to that for control cells.

Asterisks indicate significant differences relative to the control group (p < 0.05).

**Fig 3:**
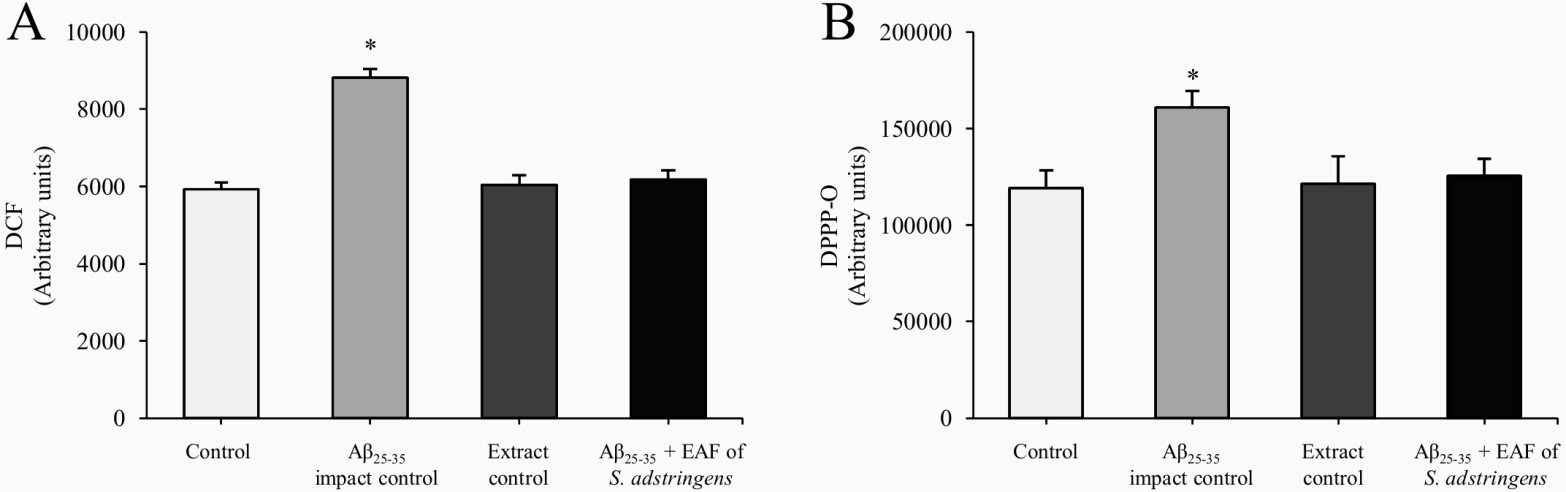
Effects of the EAF of *Stryphnodendron adstringens* on Aβ_25-35_-induced intracellular ROS accumulation and lipid peroxidation. (A) The degree of ROS generation in SH-SY5Y cells was evaluated using 2',7'-dichlorodihydrofluorescein diacetate (H_2_DCFDA) labelling. (B) The degree of lipid peroxidation in SH-SY5Y cells was evaluated using the diphenyl-1-pyrenylphosphine (DPPP) probe. Experimental treatment protocol was as the same as described in Fig 2. Results are expressed as mean fluorescence (in arbitrary units) ± SD of at least three independent experiments. Asterisks indicate significant differences relative to the control group (untreated cells) by ANOVA followed by Tukey’s test (p < 0.05), conducted in GraphPad Prism 5 software.

The degree of ROS generation in cells was measured using 2',7'-dichlorodihydrofluorescein diacetate (H_2_DCFDA) labelling. This assay provides an index of cell cytosolic oxidation [11]. The oxidative effects of Aβ_25–35_ are widely known [8,9,11,13,18] and Fig 3A shows a significant 49% increase in total ROS after exposing SH-SY5Y cells to Aβ_25–35_ for 24 h compared with the untreated control (p < 0.05), indicating an increase in cellular ROS production. Cells treated with the EAF of *S*. *adstringens* alone or as a pretreatment for 2 h prior to Aβ_25–35_ treatment showed levels of total ROS similar to the control group (increase in total ROS of only 2% and 4%, respectively), indicating that EAF of *S*. *adstringens* at a concentration of 15.62 μg/ml exerted significant inhibition of Aβ_25-35_-induced ROS accumulation. It is worth mentioning that the antiradical potential of phenolic compounds present in the EAF of *S*. *adstringens* is, undoubtedly, one of the most recognized properties of these compounds [40]. Taken together, these results corroborate the findings of other studies [11,13,18] and suggest that the EAF of *S*. *adstringens* may attenuate Aβ_25-35_-induced mitochondrial dysfunction, at least in part, by reducing ROS generation, as ROS can result in the free radical attack of membrane phospholipids, leading to mitochondrial depolarization.

Regarding lipid peroxidation, several authors have suggested that excessive ROS production can also lead to lipid peroxidation [19]. Indeed, Fig 3B shows that SH-SY5Y cells exposed to Aβ_25–35_ for 24 h exhibited a significant (35%) increase in lipid peroxidation compared with the control group (p < 0.05). Pretreatment with the EAF of *S*. *adstringens* for 2 h was able to inhibit this Aβ-induced lipid peroxidation, showing an increase in lipid peroxidation of only 5%, which was not a statistically significant difference compared to the control (p > 0.05).

### Effects of the EAF of *S*. *adstringens* on mRNA expression of AD-related genes

**Fig 4:**
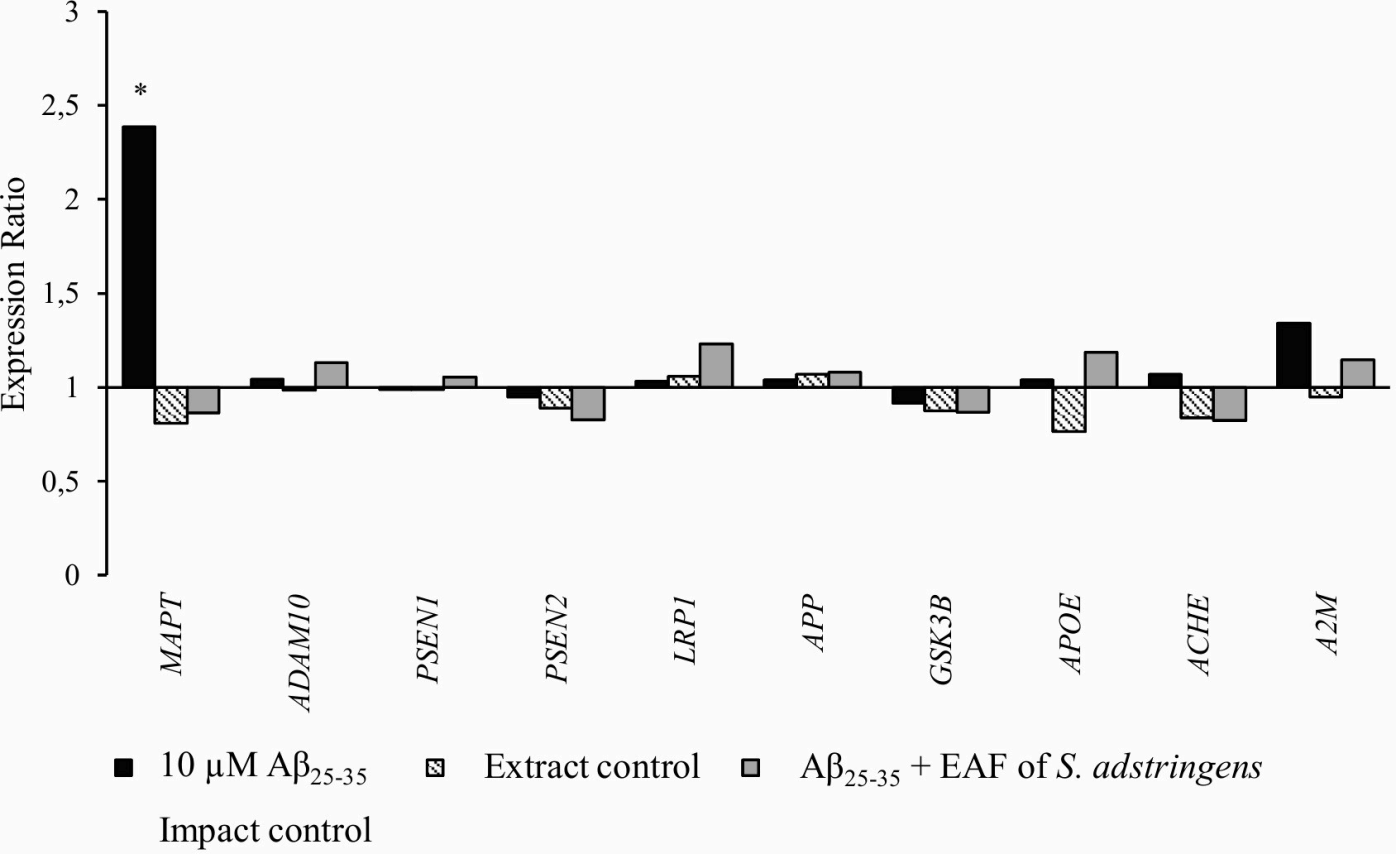
Effects of the EAF of *Stryphnodendron adstringens* on mRNA expression of AD-related genes. The quantification of mRNA was measured by RT-qPCR. Experimental treatment protocol was as the same as described in Fig 2. An Aβ impact control and an extract control were also shown. Results are expressed as expression ratio relative to reference genes of three replicates. Asterisks indicate significant differences in relative expression compared to the control using *GAPDH* and *HPRT1* as reference genes. Statistical evaluation of reference gene and target expression levels was performed using the standalone software REST 2009, with efficiency correction. Statistical difference was defined as a two-fold change in expression with a p-value < 0.05 comparing each treatment with the control. All expression levels, standard errors, 95% confidence intervals and p-values are described in S1 Table.

The mRNA expression of 10 genes related to AD was also evaluated to investigate the impact of this fraction on neuroprotection. The genes *A2M*, *ACHE*, *ADAM10*, *APOE*, *APP*, *GSK3β*, *LRP1*, *MAPT*, *PSEN1* and *PSEN2* were evaluated. The data in Fig 4 show that exposure of SH-SY5Y cells to Aβ_25–35_ significantly (p < 0.05) increased the mRNA expression ratio of the microtubule-associated protein tau (*MAPT*) gene (expression ratio > 2.0) in comparison to the control. Pretreatment with 15.62 μg/mL of the EAF of *S*. *adstringens* notably prevented overexpression of the *MAPT* mRNA gene (expression ratio of 2.387x).

The *MAPT* gene codes for the tau protein, a phosphorylated protein identified as the major component of neurofibrillary tangles that are known to be associated with the pathogenesis of AD [44]. The hyperphosphorylation of tau protein may play an important role in Aβ-induced neurodegeneration [12]. Our results suggest that the EAF of *S*. *adstringens* may protect SH-SY5Y cells by inhibiting *MAPT* gene overexpression, which is related to the deposition of intra-neuronal neurofibrillary tangles, leading to oxidative stress, cell injury and consequently neurodegeneration [44]. However, more studies, for example of proteomics, should be carried out to confirm our findings.

### Capillary electrophoresis fingerprint of the EAFs of *G*. *ulmifolia*, *L*. *brasiliense*, *P*. *cupana*, *P*. *pluviosa* and *S*. *adstringens*

It is known that the chemical composition of plant material can vary due to several factors, which can hamper the assessment of therapeutic claims [34]. Because of this, it is important to develop analytical methodologies that allow quality control of plant fractions. Previous studies have developed high-performance liquid chromatography (HPLC) fingerprints to characterize the chemical constituents of the ethanol leaf extract of *G*. *ulmifolia* [37,45] and the EAFs of *L*. *brasiliense* [21], *P*. *cupana* [26], *S*. *adstringens* [46] and *T*. *catigua* [28]. Our research group also published a methodology using capillary electrophoresis to analyse the EAF of *T*. *catigua* [32].

This work shows for the first time the electropherogram fingerprints for the EAFs of *G*. *ulmifolia*, *L*. *brasiliense*, *P*. *cupana*, *P*. *pluviosa* and *S*. *adstringens* obtained by capillary electrophoresis, which can be used for quality control of plant materials and their preparations. The chemical fingerprinting for EAF of *G*. *ulmifolia*, *L*. *brasiliense*, *P*. *cupana*, *P*. *pluviosa* and *S*. *adstringens* was performed following the steps previously described by our research group [32].

**Fig 5:**
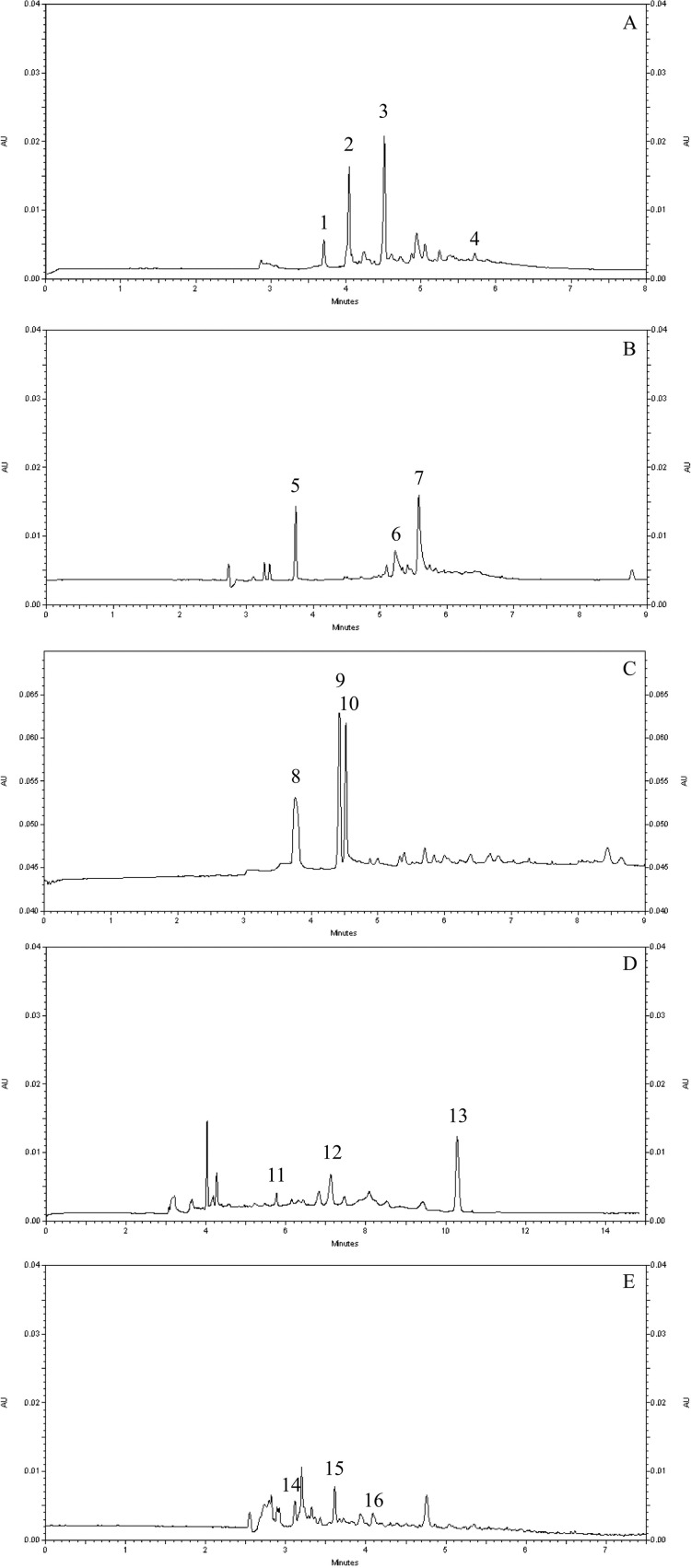
Electropherogram fingerprints of the EAFs of *Guazuma ulmifolia*, *Limonium brasiliense*, *Paullinia cupana*, *Poincianella pluviosa* and *Stryphnodendron adstringens*. The electropherograms were obtained by capillary electrophoresis under UV conditions. (A) Electropherogram of the EAF of *G*. *ulmifolia*. Peaks: 1) epiafzelechin-(4β→8)-epicatechin, 2) mixture of epigallocatechin and epicatechin, 3) procyanidin B2 (PB2), 4) procyanidin B1 (PB1). (B) Electropherogram of the EAF of *L*. *brasiliense*. Peaks: 5) epigallocatechin-3-*O*-gallate, 6) samarangenin A, 7) samarangenin B. (C) Electropherogram of the EAF of *P*. *cupana*. Peaks: 8) caffeine, 9) catechin, 10) epicatechin. (D) Electropherogram of the EAF of *P*. *pluviosa*. Peaks: 11) pyrogallol, 12) ellagic acid, 13) gallic acid. (E) Electropherogram of the EAF of *S*. *adstringens*. Peaks: 14) gallocatechin, 15) epigallocatechin, 16) PB2. Experimental conditions: uncoated fused-silica capillary, 60.2 cm (50.0 cm effective length) x 75 μm i.d.; temperature 25°C; hydrodynamic injection; UV detection at 214 nm. Detailed experimental conditions are described in Table 4. Electropherograms are shown separately in S3–S7 Figs.

Optimization of the methods was done by selecting the best capillary electrophoresis mode (capillary zone electrophoresis) and adjusting electrophoretic parameters, such as the detection wavelength, background electrolyte (BGE) characteristics (buffer concentration and pH), modifier (type and concentration of cyclodextrin, organic solvent) and sample concentration. The effects of the borate buffer concentration and pH were studied over ranges from 60 to 100 mmol/L and 8.50 to 9.00, respectively, and cyclodextrin [β-cyclodextrin (β-CD), methyl-β-cyclodextrin (M-β-CD) and 2-hydroxypropyl-β-cyclodextrin (HP-β-CD)] were screened at concentrations ranging from 10 to 20 mmol/L. Acetonitrile (10%) as an organic modifier and sample concentrations over the range from 250 to 500 μg/mL were also evaluated. The experimental conditions established and fingerprints are presented in Table 4 and Fig 5, respectively.

**Table 4:** Capillary electrophoresis experimental conditions established for chemical fingerprint evaluation of the semipurified fractions.

Experimental conditions	*Guazuma ulmifolia*	*Limonium brasiliense*	*Paullinia* *cupana*	*Poincianella* *pluviosa*	*Stryphnodendron adstringens*
Sample concentration (μg/mL)	250	250	500	500	250
Borate buffer concentration (mmol/L) pH	60 8.80	80 8.80	80 8.80	100 8.50	100 8.50
Modifier	10% acetonitrile	10 mmol/L M-β-CD	10 mmol/L HP-β-CD	10 mmol/L HP-β-CD	10 mmol/L β-CD
Voltage (kV)	30	30	25	30	30
Injection	0.5 psi; 5 s	0.5 psi; 3 s	0.5 psi; 3 s	0.5 psi; 5 s	0.5 psi; 5 s
Run time (min)	8	9	9	14.5	7.5

As we observed in another study [32], the capillary electrophoresis methodologies developed in this work were faster, less expensive and less polluting than the equivalent HPLC methods currently in use [21,26].

The chemical fingerprint qualitatively revealed a predominant amount of condensed tannins, flavonoids and phenolic acids. Some authors have suggested that polyphenols, such as astaxanthin, icariin, curcumin, resveratrol, epigallocatechin gallate, and quercetin, as well as extracts with antioxidant activity, can protect SH-SY5Y and PC12 cells against Aβ_25-35-_induced cytotoxicity by inhibiting oxidative stress, apoptosis and tau protein hyperphosphorylation; this has been shown to exert a protective effect on learning and memory abilities in rats [7,11,12,19]. The polyphenols are also recognized as potent antioxidants and are responsible for the *in vitro* anti-AChE effect [37,39,40,45]. Boasquívis et al. [42] showed that the synergic effect of the polyphenol constituents of a *P*. *cupana* extract, despite the high content of caffeine, may be responsible to reduce the Aβ aggregation and delay the Aβ-induced paralysis in *C*. *elegans* models of AD. The polyphenols are also related to a reduction in the cleavage of Aβ precursor protein and the production of Aβ [1], supporting our results suggesting the suitability of using these fractions in future studies related to AD.

### Quality control of the EAF of *S*. *adstringens*

Plant biophenols are widely known as a natural weapon against neurodegenerative disorders [7,12,18]. In our previous reports, we described the isolation and identification of various flavanols [gallocatechin, epigallocatechin, 4'-*O*-methylgallocatechin, epigallocatechin-3-*O*-gallate, epigallocatechin-3-*O*-(3,5-dimethyl)-gallate, epigallocatechin-3-*O*-(3-methoxy-4-hydroxybenzoate)] [47], prodelphinidins (epigallocatechin-gallocatechin, epigallocatechin-epigallocatechin, epigallocatechin-epigallocatechin-3-*O*-gallate, epigallocatechin-epigallocatechin-3-*O*-*p*-hydroxybenzoate, epigallocatechin-3-*O*-*p*-hydroxybenzoate) [47], prorobinetinidins [robinetinidol-epigallocatechin, robinetinidol-epigallocatechin-3-*O*-gallate, robinetinidol-gallocatechin, 4'-*O*-methylrobinetinidol-4'-*O*-methylepigallocatechin, 4'-*O*-methylrobinetinidol-4'-*O*-methylgallocatechin)] [48,49] and proanthocyanidins [epigallocatechin-3-*O*-gallate-epigallocatechin-3-*O*-gallate, epigallocatechin-epigallocatechin-3-*O*-(3,5-dimethyl)-gallate, epigallocatechin-3-*O*-(3-methoxy-4-hydroxy)-benzoate, gallocatechin-epigallocatechin-3-*O*-gallate, 4'-*O*-methylgallocatechin-4'-*O*-methylgallocatechin] [47,50] from the EAF of *S*. *adstringens*.

To perform quality control on this fraction, the Brazilian pharmacopoeia [46] recommends the determination of two flavonoids: gallic acid and gallocatechin. In fact, the Brazilian pharmacopoeia guides the production and partition of the CE of *S*. *adstringens* in the same way as we prepared it. Thus, the determination of gallic acid and gallocatechin was performed on the EAF of *S*. *adstringens* and expressed as μg/mg of CE. Gallic acid has a retention time (RT) of 10.0 min; our sample presented a gallic acid content of 34.389 ± 5.018 μg/mg of CE (y = 217642x + 373564, r^2^ = 0.9919). Gallocatechin has a retention time of 12.5 min; our sample presented a gallocatechin content of 72.451 ± 7.185 μg/mg of CE (y = 192381x + 304228, r^2^ = 0.9922).

In summary, our results suggest that possible synergy among the phytoconstituents present in the EAF of *S*. *adstringens* might be responsible for the *in vitro* protective effects described in the present study. Indeed, a study with an aqueous extract of *S*. *adstringens* with two times less TPC than our fraction (195.16 ± 0.94 mg GAE/g sample) showed that the extract increased the intracellular ROS levels, induced ΔΨm dysfunction and promoted apoptosis-induced cell death in B16F10Nex-2 melanoma cell [51], reinforcing our results.

With respect to toxicity, Costa et al. [52] showed that the EAF of *S*. *adstringens* had no genotoxic effect in mice in the range of 750 to 2.25 x 10^3^ mg/kg and showed antimutagenic activity at the dose of 750 mg/kg. By the *Artemia salina* assay, the EAF of *S*. *adstringens* showed low toxicity in the range of 10 to 1.0 x 10^3^ mg/L [52]. However, further rigorous assessments should investigate the biological effectiveness of appropriate dietary supplementation with the EAF of *S*. *adstringens*, as well as the potential toxicological aspects of this compounds in physiologically relevant AD models.

## Materials and methods

### Materials

#### Chemicals

Ultrapure water was obtained using the Milli-Q water system (Millipore, Bedford, MA, USA). Analytical grade acetone and ethyl acetate were obtained from Merck (Darmstadt, Germany) and alcohol was obtained from Panreac AppliChem (Barcelona, Spain). HPLC-grade solvents were obtained from J.T. Baker. Dulbecco’s modified Eagle’s medium (DMEM), antibiotic-antimycotic solution and foetal bovine serum (FBS) were obtained from Gibco by Life Technologies Inc. MTT was purchased from Molecular Probes (Eugene, USA). Dimethyl sulfoxide (DMSO) was obtained from Synth (Labsynth, São Paulo, Brazil). Aβ_25–35_, the genes *A2M*, *ACHE*, *ADAM10*, *APOE*, *APP*, *GSK3β*, *LRP1*, *MAPT*, *PSEN1*, *PSEN2*, *HPRT1* and *GAPDH*, β-CD, M-β-CD, HP-β-CD, DPPP, H_2_DCFDA and Rh123 were obtained from Sigma-Aldrich (St. Louis, USA). Analytical grade standards epicatechin, caffeine, catechin, ellagic acid, gallic acid (Sigma-Aldrich), pyrogallol (Fluka Analytical), gallocatechin (MP Biomedicals), epiafzelechin-(4β→8)-epicatechin, epigallocatechin, procyanidin B1 (PB1), procyanidin B2 (PB2), epigallocatechin-3-*O*-gallate, samarangenin A and samarangenin B, isolated by our research group [24,29,31], were used for peak identification. All other chemicals used were of the highest commercially available grade.

#### Plant materials

The plant materials were collected with permission from IBAMA-SISBIO (No. 11995–3, 2 November, 2010, authentication code 46367613) under the responsibility of J. C. P. Mello. All voucher species were deposited in the Herbarium of the Universidade Estadual de Maringá (HUEM). The bark of *G*. *ulmifolia* was collected in December 2004 in Ibiporã, Paraná, Brazil (23°18'15.2"S; 50°58'32.7"W). Rhizomes of *L*. *brasiliense* were collected in February 2013, in Rio Grande, Rio Grande do Sul, Brazil (31°59'33"S; 52°10'43"W). Seeds of *P*. *cupana* were obtained from Alta Floresta, Mato Grosso, Brazil (9°51'25.7"S; 56°03'58.3"W) in November 2012. The bark of *P*. *pluviosa* was collected in Maringá, Paraná, Brazil (23°24'10''S; 51°56'28''W) in January 2015. The stem bark of *S*. *adstringens* was collected in São Jerônimo da Serra, Paraná, Brazil (23°42'28''S; 50°46'26''W) in March 2008. The bark of *T*. *catigua* was obtained in May 2011 in Caetité, Bahia, Brazil (14°05'35''S; 42°34'20''W). Voucher specimens were deposited at HUEM under the numbers 10491, 27725, 9065, 12492, 14321 and 19434, respectively. The species *G*. *ulmifolia*, *P*. *cupana*, *S*. *adstringens* and *T*. *catigua* were identified by Prof. Dr. Cássia Mônica Sakuragui (Universidade Federal do Rio de Janeiro). *L*. *brasiliense* and *P*. *pluviosa* were identified by Prof. Dr. Lilian Auler Mentz (Universidade Federal do Rio Grande do Sul) and Prof. Dr. Maria Conceição de Souza (Universidade Estadual de Maringá), respectively. Acess to the botanical material was registered by the Brazilian Biodiversity System–*SisGen*—*Sistema Nacional de Gestão do Patrimônio Genético e do Conhecimento Tradicional Associado* under numbers ADA98FE, ACA171E, A06ADCB, A8B4204, 010252/2015-0 and A6DD2D2, respectively.

#### Preparation of crude extracts and semipurified fractions

The CEs and EAFs of *G*. *ulmifolia*, *L*. *brasiliense*, *P*. *cupana*, *P*. *pluviosa*, *S*. *adstringens* and *T*. *catigua* were prepared as previously described [22,23,25,27–29]. CEs and EAFs were concentrated and lyophilized before use.

### Evaluation of antioxidant activity using *in vitro* tests

All analyses were performed at least in triplicate, in 96-well microplates, and read on a Bio-tek Power Wave XS or a luminometer (Spectra max L1-Channel; Molecular Devices).

#### 2,2'-diphenyl-1-pocrylhydrazyl (DPPH) radical scavenging assay

CEs and EAFs were tested in relation to the reduction of the DPPH radical, as previously described [53]. The samples were diluted in methanol and prepared in the range of 0.78 to 25 μg/mL. Absorbance values were measured at 517 nm using a microplate spectrophotometer. Negative control, blank and positive control samples were used. A graph of the percentage of antioxidant activity versus the concentration of the extracts/fractions tested, in μg/mL, was constructed. The IC_50_ value was calculated by linear regression.

#### Ferric reducing antioxidant power (FRAP) assay

The FRAP assay was performed according to previously published methods [54]. Ethanol was used for dilution of the Trolox standard and for the preparation of different concentrations of plant extracts/fractions (15 to 60 μg/mL). Absorbance values were measured using a microplate spectrophotometer at 595 nm. To determine the total antioxidant activity of the samples, the Trolox calibration curve (20 μmol/L to 600 μmol/L, y = 0.0027x + 0.0359, r² = 0.9981) was determined and the equation of the straight line was obtained from the absorbances of the different dilutions of extracts/fractions. The results were expressed as Trolox equivalent antioxidant capacity (TEAC).

#### 2,2'-azino-bis(3-ethylbenzothiazoline-6-sulphonic acid) (ABTS) antioxidant assay

The ABTS antioxidant assay was performed according to previously published methods [55]. CEs and EAFs were evaluated at concentrations of 30 to 300 μg/mL (in ethanol). The samples were read using a microplate spectrophotometer at 734 nm. The Trolox calibration curve (40 μmol/L to 1.0 x 10^3^ μmol/L; y = -0.0005x + 0.6467; r² = 0.9961) was used to determine the total antioxidant activity and the equation of the line obtained from the absorbance values of the dilutions of the CEs/EAFs. The results were expressed as TEAC.

#### Xanthine oxidase activity assay

CEs and EAFs of the plants were diluted in 50% ethanol serially, in the range of 4.24 to 0.07 μg/mL, using a total of six concentrations. The xanthine oxidase assay was performed according to previously published methods [56], with some modifications. The addition of xanthine oxidase was performed automatically by luminometer. Blank (50% ethanol), negative control and positive control (Trolox) samples were used. A graph of the percentage of antioxidant activity *versus* the concentration of the CEs/EAFs tested was constructed in concentrations of μg/mL. The IC_50_ was calculated by linear regression.

### Determination of total phenol content using the Folin–Ciocalteu reagent method

Determination of the TPC of the CEs/EAFs was performed using Folin–Ciocalteu reagent according to previously described procedures [20,57], with a few modifications. The absorbance was read at 760 nm using a spectrophotometer (USB 2000+, Ocean Optics). Gallic acid (0.8 to 6.4 μg/mL) was used as the standard to obtain the calibration curve (y = 0.1068x + 0.006, r² = 0.9985). The TPC of the CEs/EAFs was expressed as mg of GAE/g sample.

### Evaluation of AChE inhibitory activity using a microplate AChE inhibition assay

CEs and EAFs were tested using a 96-well microplate assay based on previously published methods [15,16], with minor modifications. CE was evaluated in the range of 0.25 to 5 mg/mL and EAF at 0.05 to 1 mg/mL in ultrapure water. The reaction was started by the addition of the enzyme solution and the absorbance was monitored at 412 nm every 60 s for 10 min. The rates of reaction were calculated using appropriate software. The percentage of inhibition was calculated by comparing the rates of inhibition for the sample and the blank (ultrapure water). An inhibition curve was obtained by plotting the percentage of inhibition versus the logarithm of the inhibitor concentration in the assay solution. IC_50_ was determined from the inhibition curve by linear regression analysis. Physostigmine (Sigma-Aldrich) was used as the positive control.

### Neuroprotective effects of the EAFs of *L*. *brasiliense*, *P*. *pluviosa* and *S*. *adstringens*

#### EAF stock solution and working solution

The EAF stock solutions (4.0 x 10^4^ µg/mL) of *L*. *brasiliense*, *P*. *pluviosa* and *S*. *adstringens* were solubilized in an alcohol/cell medium mixture (70/30, v/v), sterilized by filtration and stored at 4°C. The stock solution was diluted to working concentration (4.0 x 10^3^ μg/mL) in cell medium before use.

#### Aβ_25–35_ stock solution and working solution

Aβ_25–35_ was dissolved at 1 mM in sterile distilled water. Treatments were performed with a working solution at 100 μM in cell medium supplemented with 10% FBS, to achieve a final concentration of 10 μM in each well.

#### Cell culture

Human SH-SY5Y neuroblastoma cells were kindly provided by Prof. Dr. Catarina Satie Takahashi from Universidade de São Paulo (Ribeirão Preto, Brazil) and maintained in DMEM containing 10% FBS in a humidified atmosphere of 5.0% CO_2_ in air at 37.0°C, as previously described [58]. Experiments were performed at 80% confluence.

#### Determination of cell viability

Cell viability was assessed using a conventional MTT reduction assay as described previously [59], with some modifications. The assay was performed with six replicates. Cells at a density of 2.0 x 10^4^ cells per well were placed in 96-well plates with 100 μL of fresh medium supplemented with 10% of FBS. After 24 h of stabilization, the cells were pretreated with three different concentrations of EAFs (7.81, 15.62 and 31.25 μg/mL, solubilized in DMEM plus 10% FBS) for 2 h. After 2 h, the treatment was combined with 10 μM Aβ_25–35_ and incubated for another 24 h at 37.0°C in 5.0% CO_2_. A solvent control condition (DMEM + 10% FBS) was used as a control for the statistical analysis. An Aβ impact control (10 μM Aβ_25–35_ solubilized in DMEM plus 10% FBS) and an extract control (each type of EAF at each test concentration + DMEM + 10% FBS) were also included. After the treatment-associated period, the culture medium was discarded and 100 μL of MTT (500 μg/mL) was added to all wells and the plates were incubated for 4 h. The MTT solution was then removed and 100 μL of DMSO was added to all wells to dissolve the dark blue crystals. The plates were shaken for a few minutes and read on a Thermo Plate reader (Thermo Plate, China) using a wavelength of 540 nm. Data were analysed and expressed as percentages relative to the control. The pretreatment and treatment were performed based on previous studies [11,18,58].

### Evaluation of the protective effects of the EAF of *S*. *adstringens*

#### Mitochondrial membrane potential (ΔΨm) assay

ΔΨm was evaluated during the exposure of SH-SY5Y cells to the EAF of *S*. *adstringens* at 15.62 μg/mL for 2 h, then combined with 10 μM Aβ_25–35_ for another 24 h, using the Rh123 probe. Afterward, the cells were incubated with 5 μg/mL Rh123 for 15 min to verify ΔΨm. A solvent control (DMEM + 10% FBS) was used as a control for the statistical analysis. An Aβ impact control (10 μM Aβ_25–35_ solubilized in DMEM plus 10% FBS) and an extract control (EAF of *S*. *adstringens* at 15.62 μg/mL + DMEM + 10% FBS) were also included. The data acquisition and analysis were performed using a FACSCalibur flow cytometer equipped with CellQuest software. A total of 1.0 x 10^4^ events were acquired in the region corresponding to the cells. Alterations in Rh123 fluorescence were quantified using an index of variation (IV) obtained from the equation (MT—MC)/MC, in which MT is the median fluorescence for the treated cells and MC is the median fluorescence for the control cells. Negative IV values correspond to depolarization of the mitochondrial membrane [60].

#### Fluorimetric detection of ROS production

The production of ROS was evaluated during the exposure of SH-SY5Y cells to the EAF of *S*. *adstringens* at 15.62 μg/mL for 2 h, then combined with 10 μM Aβ_25–35_ and incubated for another 24 h, using the 2',7'-dichlorodihydrofluorescein diacetate (H_2_DCFDA) probe, measuring its oxidation to the fluorescent product 2',7'-dichlorofluorescein (DCF). A solvent control, an Aβ impact control and an extract control were also included. Cells were loaded with 10 μM H_2_DCFDA and stored in the dark for 45 min and fluorescence was determined using a VICTOR *X3* spectrofluorometer at λ_ex_ = 488 nm and λ_em_ = 530 nm [61].

#### Lipid peroxidation assay

The extent of lipid peroxidation was evaluated during the exposure of SH-SY5Y cells to the EAF of *S*. *adstringens* at 15.62 μg/mL for 2 h, then combined with 10 μM Aβ_25–35_ and incubated for another 24 h, using the diphenyl-1-pyrenylphosphine (DPPP) probe. For this, cells were loaded with 50 μM DPPP for 15 min at 22°C and fluorescence was determined in a fluorescence microplate reader (VICTOR *X3*, PerkinElmer) at λ_ex_ = 355 nm and λ_em_ = 460 nm. DPPP is essentially non-fluorescent until it is oxidized to a phosphine oxide (DPPP-O) by peroxides [62]. A solvent control condition, an Aβ impact control and an extract control were also included.

### Quantification of mRNA using real-time quantitative polymerase chain reaction (RT-qPCR)

3.0 x 10^5^ cells were inoculated in a 24-well plate with 500 μL of DMEM plus 10% FBS for 24 h at 37.0°C with 5.0% CO_2_ for stabilization. Then, the cells were pretreated with the EAF of *S*. *adstringens* at 15.62 μg/mL for 2 h. After 2 h, the treatment was combined with 10 μM Aβ_25–35_ and incubated for another 24 h at 37.0°C in 5.0% CO_2_. A solvent control condition, an Aβ impact control and an extract control were also included.

#### RNA isolation and cDNA synthesis

Total RNA was isolated using a Qiagen RNeasy Minikit (Hilden, Germany), according to the manufacturer’s instructions. The purity and concentration of isolated RNA were determined by a NanoDrop Lite spectrophotometer (Thermo Scientific) and RNA integrity and quality were verified by denaturing agarose gel electrophoresis according to previously published methods [63]. cDNA synthesis of each sample was performed in triplicate using a Veriti Thermal Cycler (Applied Biosystems) with 250 ng of total RNA diluted in a final volume of 16 μL containing oligo dT (80 pmol), random primers (100 pmol) and dNTPs (0.5 mM). This first reaction mix was incubated for 10 min at 65°C. Each reaction was then thermal shocked on ice and 4 μL of a second reaction mix was added [1.3 μL of DEPC-treated H_2_O, 2 μL of Buffer 10X, 0.6 μL of MgCl_2_ (50 mM), 0.05 μL of RNase Out (Invitrogen), 0.05 μL of SuperScript III enzyme (Invitrogen)]. The final solutions were submitted to incubation at 37.0°C for 50 min to allow for cDNA synthesis and a final period of enzyme inactivation at 70.0°C for 15 min.

#### Quantitative PCR (qPCR)

The qPCR reactions were also performed in triplicate on a CFX96 Real-Time System (Bio-Rad) using 5 μL of SsoAdvanced SYBR Green Supermix (Bio-Rad), 1 μL of each oligonucleotide primer (10 pmol/μL) and 5 μL of cDNA (50 ng/μL) (1:10 dilution of input RNA). The reaction conditions were: pre-incubation at 50°C for 2 min (UDG incubation), initial denaturation at 95°C for 5 min, followed by 45 cycles of 20 s at 95°C, 30 s at 60°C and 20 s at 72°C. A melt curve analysis ranging from 50°C to 98°C was performed at the end of the reaction with 5 s of reading at every 0.5°C. The software CFX Manager 3.1 (Bio-Rad) was used to collect the data and the efficiency of the reactions was calculated in LinRegPCR software [64,65]. *HPRT1* and *GAPDH* were used as reference genes. The target genes were as follows: *A2M*, *ACHE*, *ADAM10*, *APOE*, *APP*, *GSK3β*, *LRP1*, *MAPT*, *PSEN1* and *PSEN2*.

### Development of the capillary electrophoresis fingerprint

#### Capillary electrophoresis under UV conditions

Analytical development was carried out using the Beckman P/ACE MDQ electrophoresis system (Beckman-Coulter) equipped with a filter-based UV/Vis detector and 32 Karat version 7.0 software. Fused-silica capillaries (Beckman Coulter) were used with the following dimensions: 60.2 cm total length, 50.0 cm effective length, 363 μm o.d. and 75 μm i.d. The samples were injected hydrodynamically and all electropherograms were recorded at 214 nm. The cartridge coolant was set at 25°C. The BGE conditions were set for each sample.

#### Preparation of the solution for analysis

An appropriate amount of each EAF was weighed, dissolved in 10.0 mL of a 20% methanol solution and eluted through a solid-phase extraction (SPE) cartridge (Strata C18-E, Phenomenex), as described previously [32]. All solutions for analysis were filtered through a 0.45 μm Millipore filter.

### Quality control of the EAF of *S*. *adstringens*

The gallic acid and gallocatechin content present in the EAF of *S*. *adstringens* was determined by HPLC, according to the methodology described by the Brazilian pharmacopoeia [46]. The analysis was performed with three replicates in a system consisting of a Thermo HPLC equipped with pumps and an integral degasser (Finnigan Surveyor LC Pump Plus), PDA spectrophotometric detector module (Finnigan Surveyor PDA Plus Detector), controller software (Chromquest) and autosampler (Finnigan Surveyor Autosampler Plus) equipped with a 10 μL loop for injection. For quantification, the standard curves of gallic acid and gallocatechin were constructed. The results are expressed as μg/mg of CE.

### Statistical analysis

Data are presented as the mean ± standard deviation (SD) of three replicates for each experiment. One-way analysis of variance (ANOVA) was performed to detect significant differences between samples. Statistically significant differences were defined as a p-value < 0.05. The chemometric tools used were PCA and HCA, implemented in Statistica v. 13.3 software (TIBCO Software Inc., Palo Alto, CA, USA). First, the dataset was autoscaled (transformation into z-scores) and PCA was applied to distinguish the samples according to the levels of DPPH, ABTS, FRAP, xanthine oxidase system, AChE inhibitory activity and TPC [37]. HCA was performed on the basis of Euclidean distance and Ward’s method was used to cluster the samples. Levene’s test was carried out to check for homogeneity of variance. ANOVA and the non-parametric multiple comparison Kruskal–Wallis test were used to identify noted differences among the clusters. Fisher’s least significant difference (LSD) *post hoc* multiple comparison test was applied to identify the differences observed among clusters [37]. For the MTT, ΔΨm, total ROS and lipid peroxidation assays described previously, the data were analysed using one- and two-way ANOVA with significant differences among means identified by the Tukey or Bonferroni *post hoc* tests, respectively. The statistical analyses were performed using Prism 5 software (GraphPad, San Diego, CA, USA). Statistical validation of reference gene and gene expression levels was undertaken in the stand-alone software *REST* 2009 (*Relative Expression Software Tool*/Qiagen), with efficiency correction, using a previously described method [66]. Statistically significant differences were defined as a two-fold change and a p-value < 0.05, comparing each treatment with the control.

## Conclusion

The present study provides information concerning the *in vitro* protective effects of *G*. *ulmifolia*, *L*. *brasiliense*, *P*. *cupana*, *P*. *pluviosa*, *S*. *adstringens* and *T*. *catigua* against neurodegenerative disease, specifically Alzheimer’s disease (AD). PCA and HCA were found to be useful chemometric tools for rationalizing the choice of extracts/fractions with the greatest potential to be evaluated against AD, avoiding the use of animals and allowing the optimization of time and resources invested in the research. This is the first study to use SH-SY5Y cell culture assays showing that the EAFs of *S*. *adstringens*, *P*. *pluviosa* and *L*. *brasiliense* are able to protect human neuroblastoma cells against damage induced by Aβ_25–35_. The neuroprotective effect of the EAF of *S*. *adstringens* was due, at least in part, to protection against mitochondrial depolarization, superoxide production and Aβ-induced lipid peroxidation and inhibition of *MAPT* mRNA overexpression, which suggests a decrease in the hyperphosphorylation of tau protein. The synergy between the polyphenols present in this fraction may be responsible for the biological activity observed *in vitro*. However, further rigorous studies should be conducted to investigate the biological effectiveness and the potential toxicological aspects of the EAF of *S*. *adstringens* in physiologically relevant AD models with a view to confirming and expanding our findings. The neuroprotective potential of the EAFs of *L*. *brasiliense* and *P*. *pluviosa* should also be investigated in future studies. With respect to analytical development, the developed methodologies are faster, more economical and less polluting than the equivalent methods previously established by HPLC and can be adopted for quality control routine after adequate validation.

## Supporting information

S1 Fig Principal component analysis scatter plots on the main sources of variability between the evaluated CEs/EAFs. (A) *Scores* plot (PC1 versus PC2) and (B) *loading* plot (PC1 versus PC2). Principal component analysis (PCA) was applied to distinguish the samples according to the levels of DPPH, ABTS, FRAP, xanthine oxidase system, AChE inhibitory activity and TPC. Dataset was autoscaled (transformation into z-scores) and PCA was conducted in Statistica v. 13.3 software. Note: I (CE of *Guazuma ulmifolia*); II (CE of *Limonium brasiliense*); III (CE of *Paullinia cupana*); IV (CE of *Poincianella pluviosa*); V (CE of *Stryphnodendron adstringens*); VI (CE of *Trichilia catigua*); VII (EAF of *Guazuma ulmifolia*); VIII (EAF of *Limonium brasiliense*); IX (EAF of *Paullinia cupana*); X (EAF of *Poincianella pluviosa*); XI (EAF of *Stryphnodendron adstringens*); XII (EAF of *Trichilia catigua*).
(TIF)

S2 Fig Effects of the EAFs of *Limonium brasiliense*, *Poincianella pluviosa* and *Stryphnodendron adstringens* on SH-SY5Y cells viability. SH-SY5Y neuroblastoma cells were pretreated with different concentrations (7.81 to 1.0 x 10^3^ μg/mL) of EAF of *L*. *brasiliense* (A), EAF of *P*. *pluviosa* (B) and EAF of *S*. *adstringens* (C) for 24 h. Cell viability was measured by MTT reduction assay. Data are presented as mean ± SD of six replicates. Asterisks indicates statistically significant differences between the multiple conditions comparison by ANOVA followed by Tukey’s test (p < 0.05), conducted in GraphPad Prism 5 software.
(TIF)

S3 Fig Electropherogram fingerprint of the EAF of *Guazuma ulmifolia*. Peaks: 1) epiafzelechin-(4β→8)-epicatechin, 2) mixture of epigallocatechin and epicatechin, 3) procyanidin B2 (PB2), 4) procyanidin B1 (PB1). Experimental conditions: 60 mmol/L borate buffer at pH 8.80 with 10% acetonitrile; uncoated fused-silica capillary, 60.2 cm (50.0 cm effective length) x 75 μm i.d.; temperature 25°C; hydrodynamic injection 0.5 psi x 5 s; voltage 30 kV; UV detection at 214 nm; EAF of *G*. *ulmifolia* 250 μg/mL.
(TIF)

S4 Fig Electropherogram fingerprint of the EAF of *Limonium brasiliense*. Peaks: 5) epigallocatechin-3-*O*-gallate, 6) samarangenin A, 7) samarangenin B. Experimental conditions: 80 mmol/L borate buffer at pH 8.80 with 10 mmol/L M-β-CD; uncoated fused-silica capillary, 60.2 cm (50.0 cm effective length) x 75 μm i.d.; temperature 25°C; hydrodynamic injection 0.5 psi x 3 s; voltage 30 kV; UV detection at 214 nm; EAF of *L*. *brasiliense* 250 μg/mL.
(TIF)

S5 Fig Electropherogram fingerprint of the EAF of *Paullinia cupana*. Peaks: 8) caffeine, 9) catechin, 10) epicatechin. Experimental conditions: 80 mmol/L borate buffer at pH 8.80 with 10 mmol/L HP-β-CD; uncoated fused-silica capillary, 60.2 cm (50.0 cm effective length) x 75 μm i.d.; temperature 25°C; hydrodynamic injection 0.5 psi x 3 s; voltage 25 kV; UV detection at 214 nm; EAF of *P*. *cupana* 500 μg/mL.
(TIF)

S6 Fig Electropherogram fingerprint of the EAF of *Poincianella pluviosa*. Peaks: 11) pyrogallol, 12) ellagic acid, 13) gallic acid. Experimental conditions: 100 mmol/L borate buffer at pH 8.50 with 10 mmol/L HP-β-CD; uncoated fused-silica capillary, 60.2 cm (50.0 cm effective length) x 75 μm i.d.; temperature 25°C; hydrodynamic injection 0.5 psi x 5 s; voltage 30 kV; UV detection at 214 nm; EAF of *P*. *pluviosa* 500 μg/mL.
(TIF)

S7 Fig Electropherogram fingerprint of the EAF of *Stryphnodendron adstringens*. Peaks: 14) gallocatechin, 15) epigallocatechin, 16) PB2. Experimental conditions: 100 mmol/L borate buffer at pH 8.50 with 10 mmol/L β-CD; uncoated fused-silica capillary, 60.2 cm (50.0 cm effective length) x 75 μm i.d.; temperature 25°C; hydrodynamic injection 0.5 psi x 5 s; voltage 30 kV; UV detection at 214 nm; EAF of *S*. *adstringens* 250 μg/mL.
(TIF)

S1 Table Data analysis of mRNA expression of Alzheimer’s disease-related genes using the standalone software REST 2009 with efficiency correction. All expression levels, standard errors, 95% confidence index intervals and p-values are described.
(DOCX)
