# Supplementary figures and images for: In vitro evaluation of the protective effects of plant extracts against amyloid-beta peptide-induced toxicity in human neuroblastoma SH-SY5Y cells
Source: PLoS One. 2019 Feb 14;14(2):e0212089. doi: 10.1371/journal.pone.0212089 (PMC6375598; doi:10.1371/journal.pone.0212089)

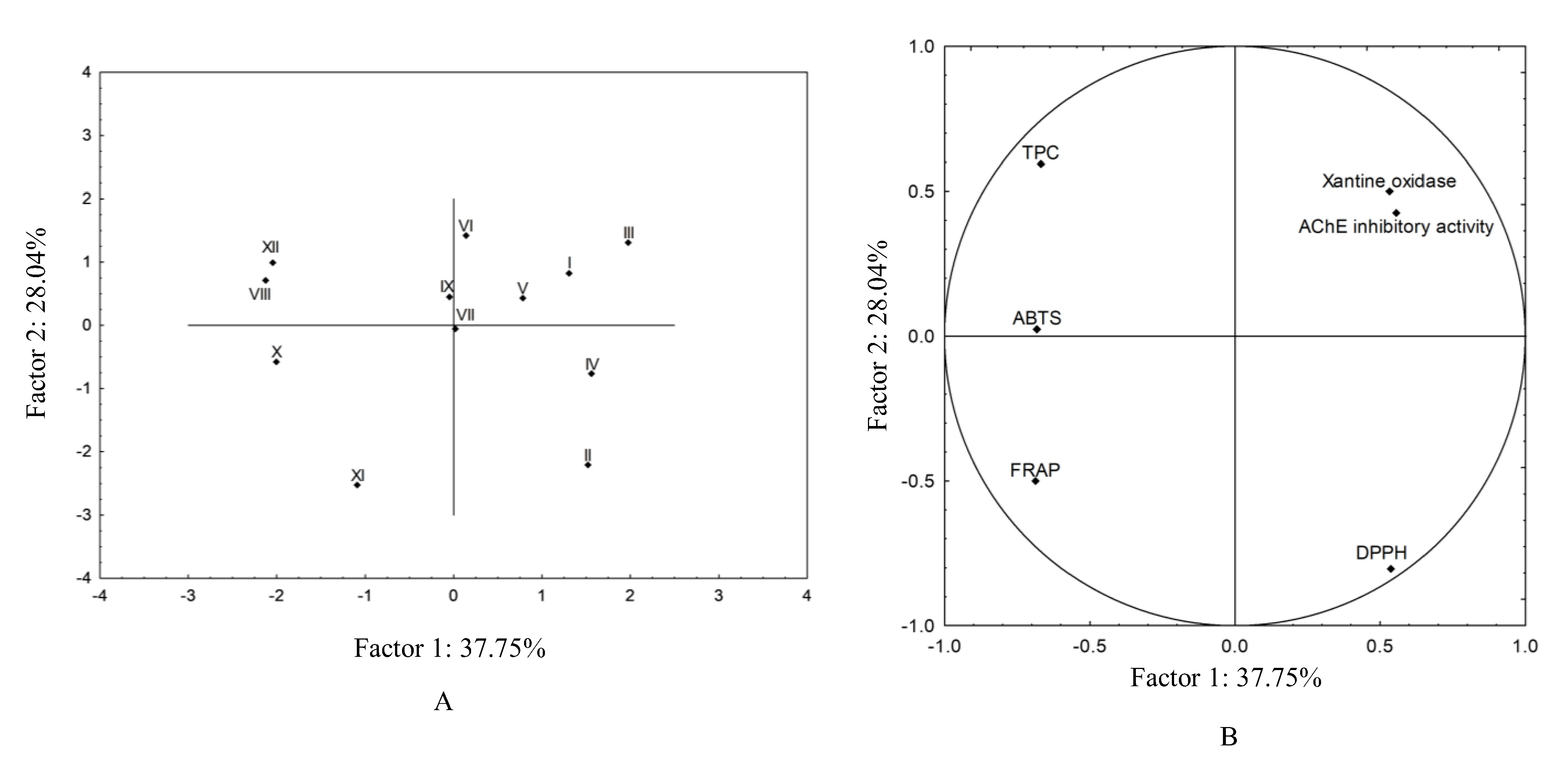

Supplement: S1 Fig — (A) Scores plot (PC1 versus PC2) and (B) loading plot (PC1 versus PC2). Principal component analysis (PCA) was applied to distinguish the samples according to the levels of DPPH, ABTS, FRAP, xanthine oxidase system, AChE inhibitory activity and TPC. Dataset was autoscaled (transformation into z-scores) and PCA was conducted in Statistica v. 13.3 software. Note: I (CE of Guazuma ulmifolia); II (CE of Limonium brasiliense); III (CE of Paullinia cupana); IV (CE of Poincianella pluviosa); V (CE of Stryphnodendron adstringens); VI (CE of Trichilia catigua); VII (EAF of Guazuma ulmifolia); VIII (EAF of Limonium brasiliense); IX (EAF of Paullinia cupana); X (EAF of Poincianella pluviosa); XI (EAF of Stryphnodendron adstringens); XII (EAF of Trichilia catigua). (TIF) [file pone.0212089.s001.tif]

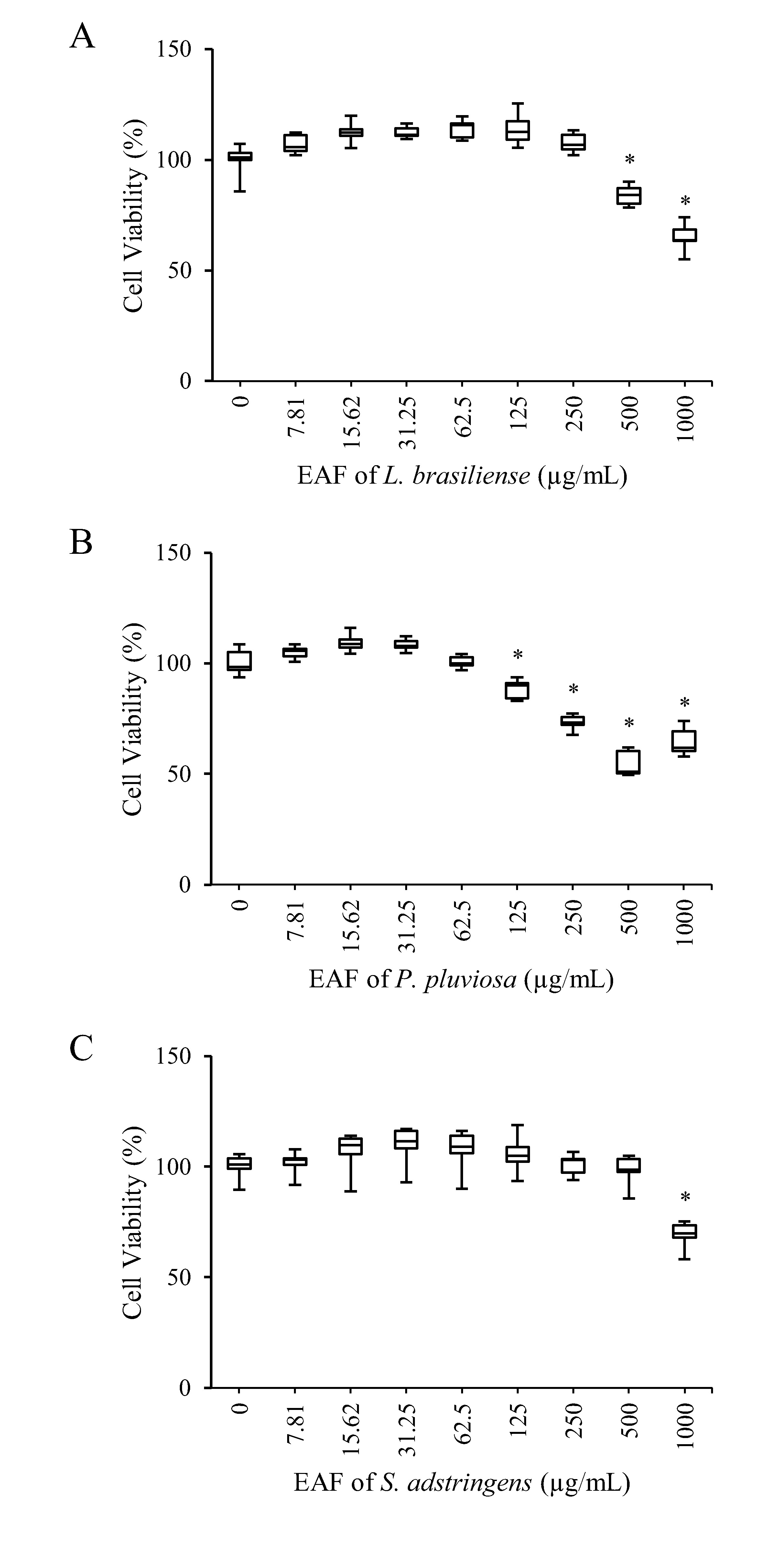

Supplement: S2 Fig — SH-SY5Y neuroblastoma cells were pretreated with different concentrations (7.81 to 1.0 x 103 μg/mL) of EAF of L. brasiliense (A), EAF of P. pluviosa (B) and EAF of S. adstringens (C) for 24 h. Cell viability was measured by MTT reduction assay. Data are presented as mean ± SD of six replicates. Asterisks indicates statistically significant differences between the multiple conditions comparison by ANOVA followed by Tukey’s test (p < 0.05), conducted in GraphPad Prism 5 software. (TIF) [file pone.0212089.s002.tif]

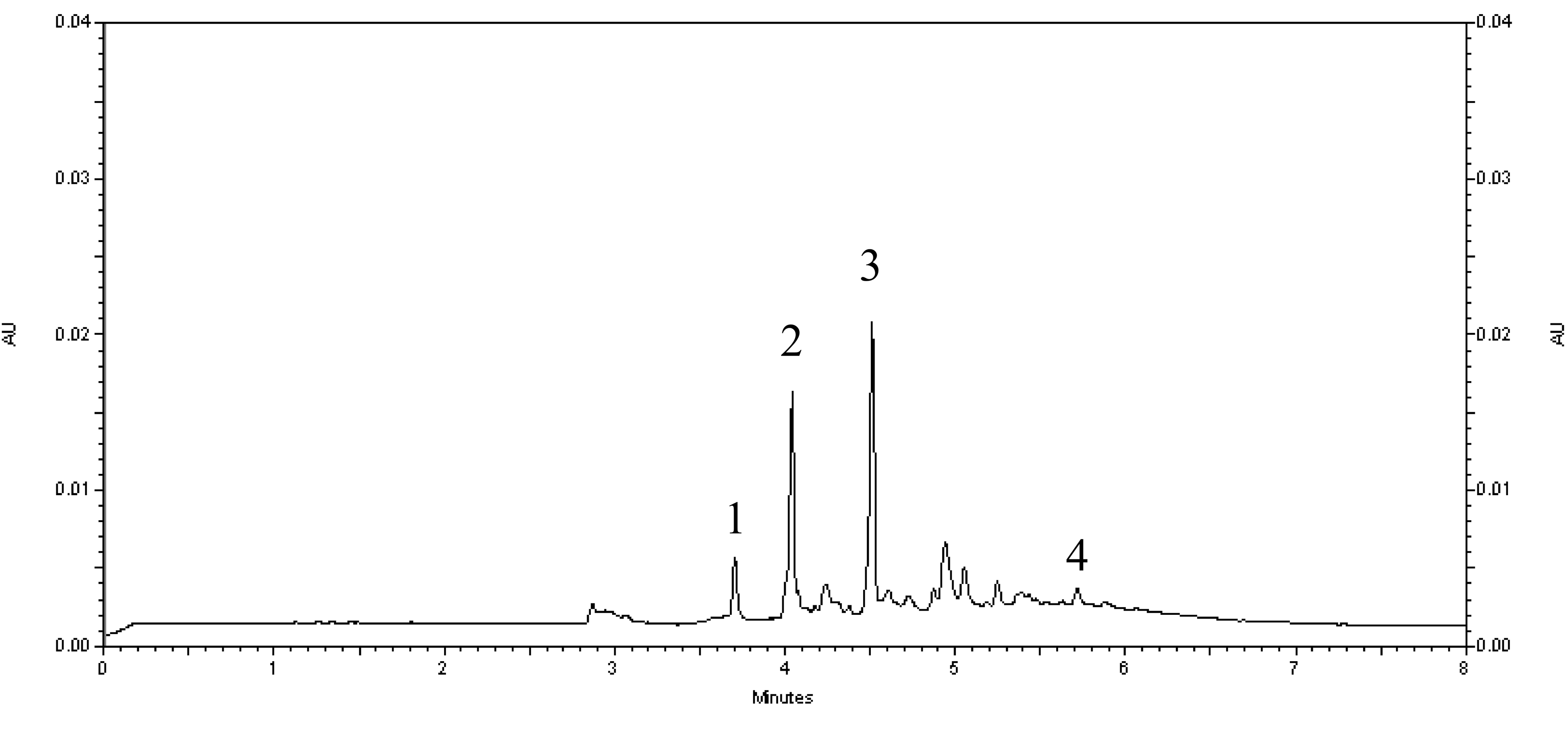

Supplement: S3 Fig — Peaks: 1) epiafzelechin-(4β→8)-epicatechin, 2) mixture of epigallocatechin and epicatechin, 3) procyanidin B2 (PB2), 4) procyanidin B1 (PB1). Experimental conditions: 60 mmol/L borate buffer at pH 8.80 with 10% acetonitrile; uncoated fused-silica capillary, 60.2 cm (50.0 cm effective length) x 75 μm i.d.; temperature 25°C; hydrodynamic injection 0.5 psi x 5 s; voltage 30 kV; UV detection at 214 nm; EAF of G. ulmifolia 250 μg/mL. (TIF) [file pone.0212089.s003.tif]

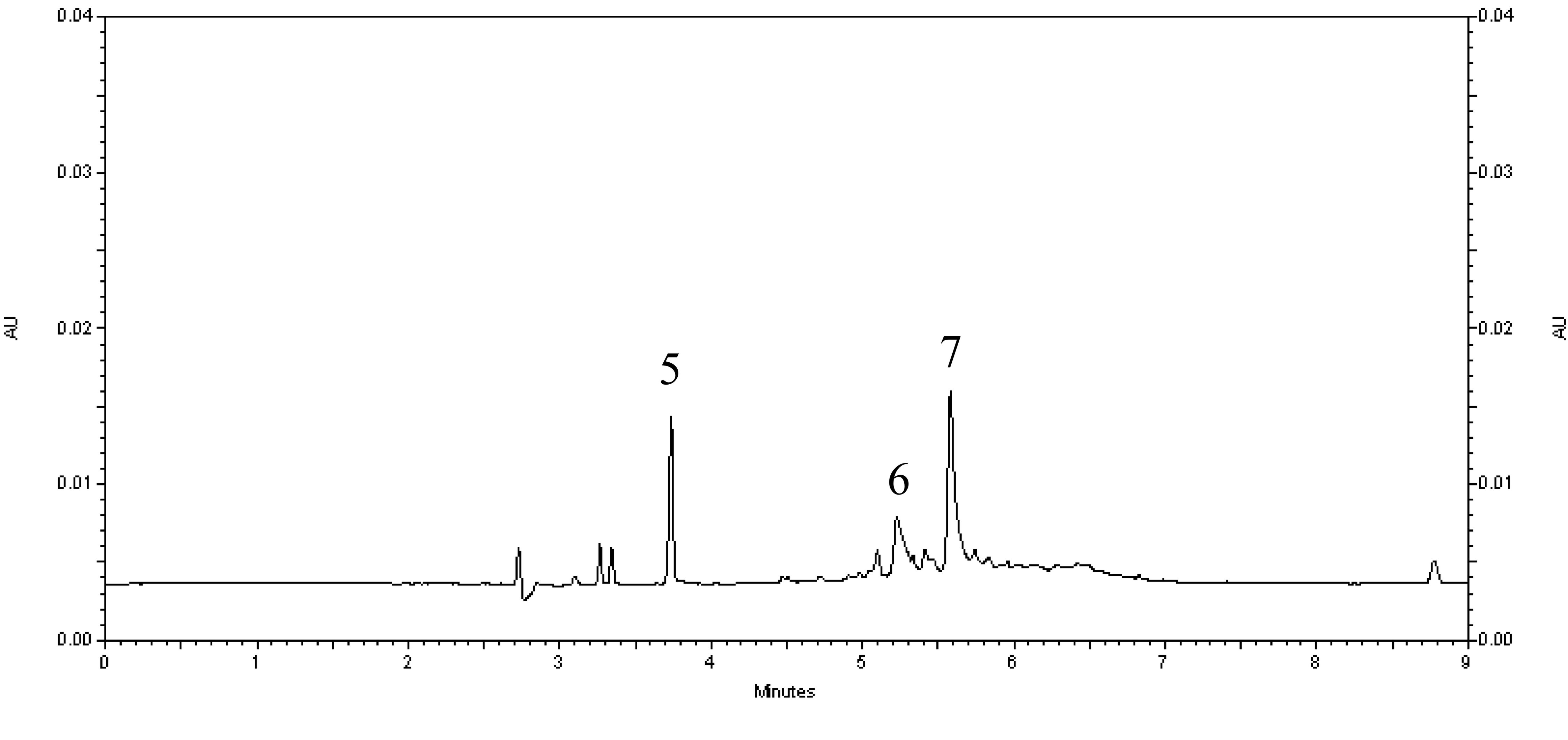

Supplement: S4 Fig — Peaks: 5) epigallocatechin-3-O-gallate, 6) samarangenin A, 7) samarangenin B. Experimental conditions: 80 mmol/L borate buffer at pH 8.80 with 10 mmol/L M-β-CD; uncoated fused-silica capillary, 60.2 cm (50.0 cm effective length) x 75 μm i.d.; temperature 25°C; hydrodynamic injection 0.5 psi x 3 s; voltage 30 kV; UV detection at 214 nm; EAF of L. brasiliense 250 μg/mL. (TIF) [file pone.0212089.s004.tif]

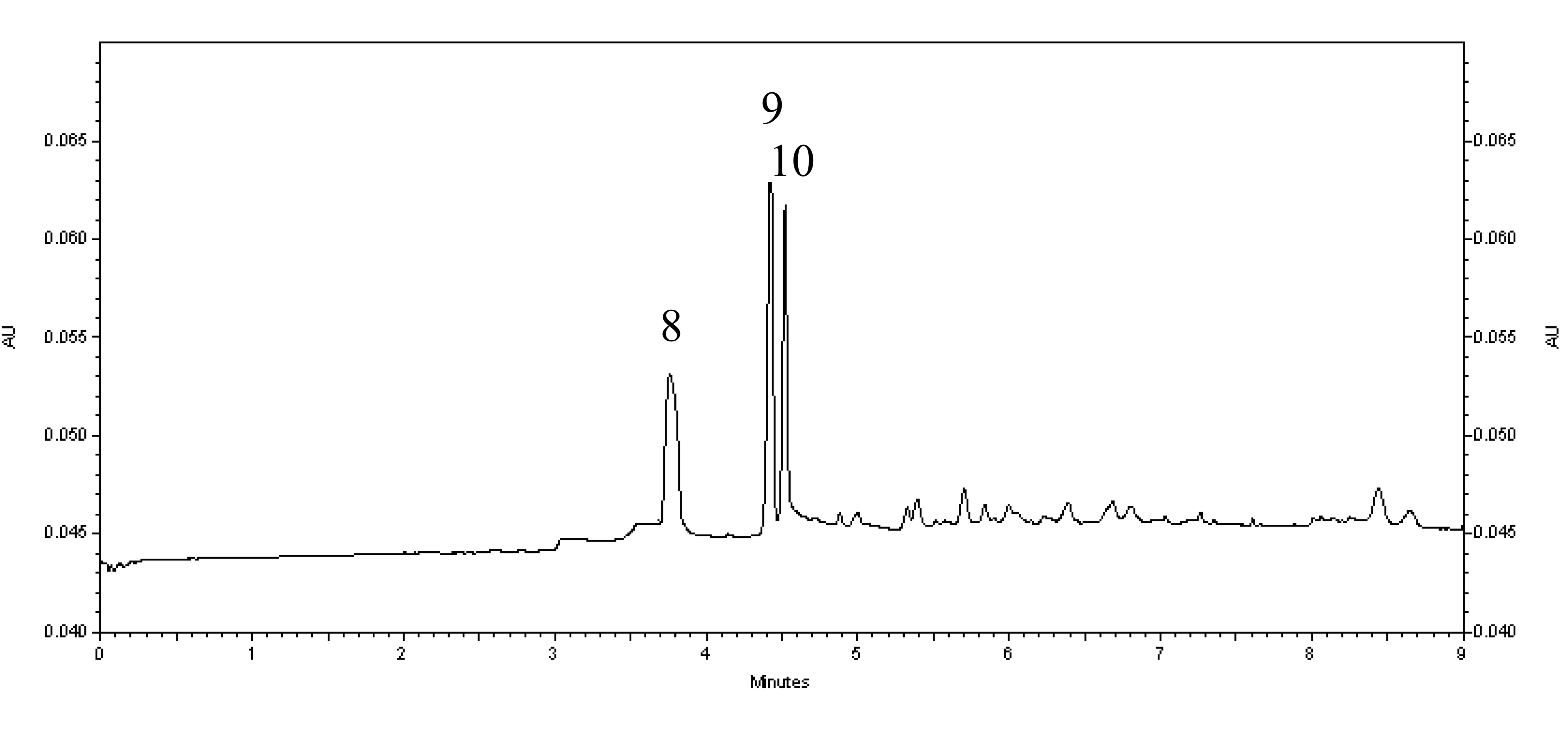

Supplement: S5 Fig — Peaks: 8) caffeine, 9) catechin, 10) epicatechin. Experimental conditions: 80 mmol/L borate buffer at pH 8.80 with 10 mmol/L HP-β-CD; uncoated fused-silica capillary, 60.2 cm (50.0 cm effective length) x 75 μm i.d.; temperature 25°C; hydrodynamic injection 0.5 psi x 3 s; voltage 25 kV; UV detection at 214 nm; EAF of P. cupana 500 μg/mL. (TIF) [file pone.0212089.s005.tif]

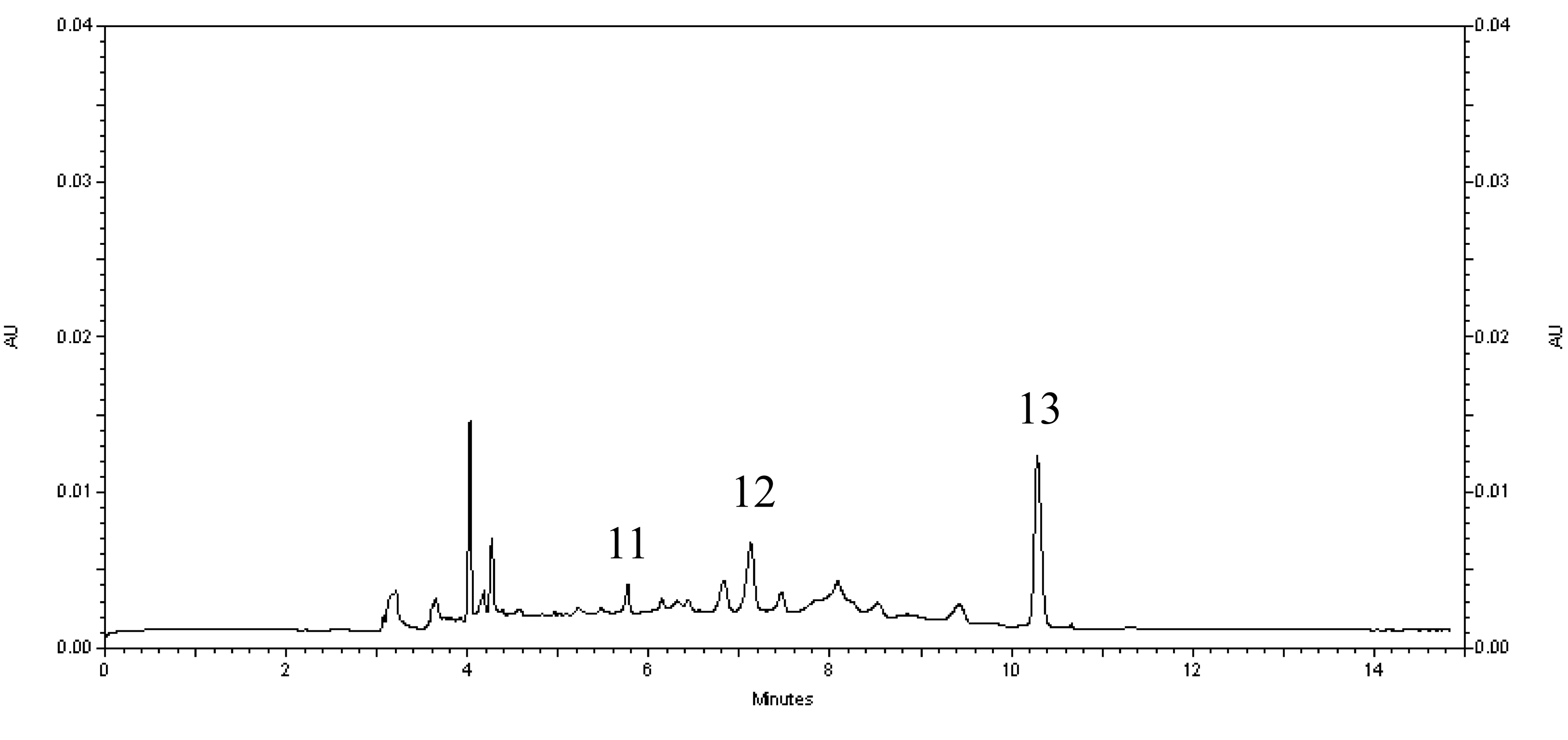

Supplement: S6 Fig — Peaks: 11) pyrogallol, 12) ellagic acid, 13) gallic acid. Experimental conditions: 100 mmol/L borate buffer at pH 8.50 with 10 mmol/L HP-β-CD; uncoated fused-silica capillary, 60.2 cm (50.0 cm effective length) x 75 μm i.d.; temperature 25°C; hydrodynamic injection 0.5 psi x 5 s; voltage 30 kV; UV detection at 214 nm; EAF of P. pluviosa 500 μg/mL. (TIF) [file pone.0212089.s006.tif]

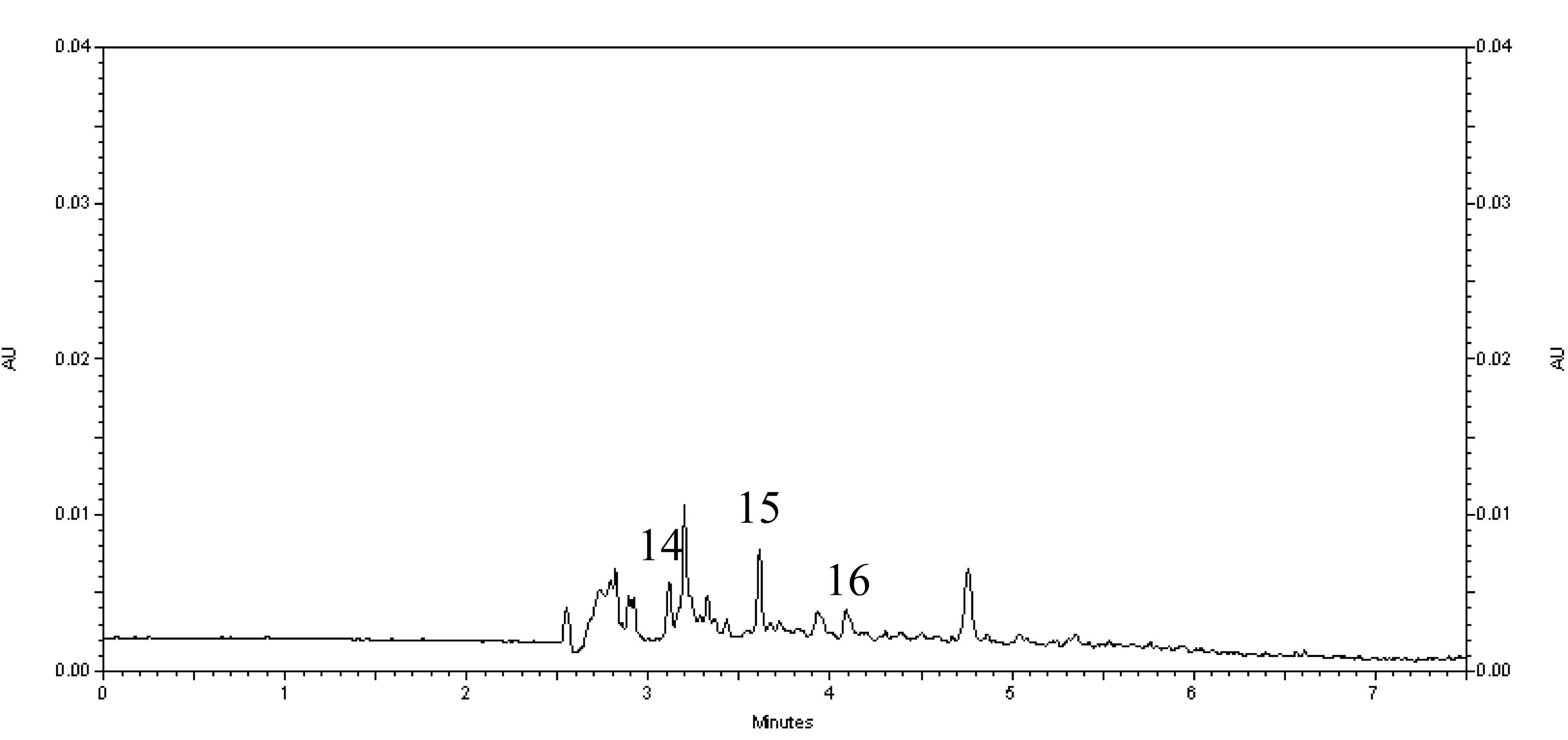

Supplement: S7 Fig — Peaks: 14) gallocatechin, 15) epigallocatechin, 16) PB2. Experimental conditions: 100 mmol/L borate buffer at pH 8.50 with 10 mmol/L β-CD; uncoated fused-silica capillary, 60.2 cm (50.0 cm effective length) x 75 μm i.d.; temperature 25°C; hydrodynamic injection 0.5 psi x 5 s; voltage 30 kV; UV detection at 214 nm; EAF of S. adstringens 250 μg/mL. (TIF) [file pone.0212089.s007.tif]
