# Supplementary material for: In vitro evaluation of the protective effects of plant extracts against amyloid-beta peptide-induced toxicity in human neuroblastoma SH-SY5Y cells
Source: PLoS One. 2019 Feb 14;14(2):e0212089. doi: 10.1371/journal.pone.0212089 (PMC6375598; doi:10.1371/journal.pone.0212089)
Supplement: S1 Table — All expression levels, standard errors, 95% confidence index intervals and p-values are described. (DOCX) [file pone.0212089.s008.docx]

**S1 Table. Data analysis of mRNA expression of Alzheimer’s disease related-genes using the standalone software REST 2009 with efficiency correction.**

| **Gene** | **Condition** | **Type** | **Reaction Efficiency** | **Expression** | **Std. Error** | **95% C.I.** | **p(H1)** | **Result (p < 0.05)** |
| --- | --- | --- | --- | --- | --- | --- | --- | --- |
|  | 10 µM Aβ_25-35_ | TRG | 1 | 2.387 | 1.239 - 5.194 | 0.683 - 9.669 | 0.024 | UP |
| *MAPT* | Control | TRG | 1 | 0.807 | 0.383 - 1.666 | 0.244 - 2.625 | 0.484 |  |
|  | Aβ_25-35_ + Stryphnodendron *adstringens* | TRG | 1 | 0.865 | 0.295 - 3.416 | 0.018 - 4.966 | 0.95 |  |
|  | 10 µM Aβ_25-35_ | TRG | 1 | 1.045 | 0.804 - 1.348 | 0.638 - 1.606 | 0.706 |  |
| *ADAM10* | Control | TRG | 1 | 0.983 | 0.770 - 1.222 | 0.670 - 1.460 | 0.861 |  |
|  | Aβ_25-35_ + Stryphnodendron *adstringens* | TRG | 1 | 1.13 | 0.936 - 1.378 | 0.884 - 1.499 | 0.119 |  |
|  | 10 µM Aβ_25-35_ | TRG | 0.956 | 0.988 | 0.710 - 1.303 | 0.592 - 1.406 | 0.929 |  |
| *PSEN1* | Control | TRG | 0.956 | 0.987 | 0.686 - 1.366 | 0.524 - 1.708 | 0.931 |  |
|  | Aβ_25-35_ + Stryphnodendron *adstringens* | TRG | 0.956 | 1.054 | 0.774 - 1.366 | 0.622 - 1.522 | 0.691 |  |
|  | 10 µM Aβ_25-35_ | TRG | 0.979 | 0.947 | 0.751 - 1.169 | 0.603 - 1.325 | 0.629 |  |
| *PSEN2* | Control | TRG | 0.979 | 0.89 | 0.698 - 1.097 | 0.550 - 1.370 | 0.317 |  |
|  | Aβ_25-35_ + Stryphnodendron *adstringens* | TRG | 0.979 | 0.825 | 0.653 - 1.008 | 0.548 - 1.077 | 0.044 | DOWN |
|  | 10 µM Aβ_25-35_ | TRG | 1 | 1.031 | 0.561 - 2.039 | 0.402 - 2.374 | 0.903 |  |
| *LRP1* | Control | TRG | 1 | 1.058 | 0.627 - 1.807 | 0.475 - 2.120 | 0.802 |  |
|  | Aβ_25-35_ + Stryphnodendron *adstringens* | TRG | 1 | 1.231 | 0.700 - 1.988 | 0.586 - 2.296 | 0.361 |  |
|  | 10 µM Aβ_25-35_ | TRG | 1 | 1.039 | 0.852 - 1.291 | 0.696 - 1.451 | 0.695 |  |
| *APP* | Control | TRG | 1 | 1.069 | 0.912 - 1.367 | 0.718 - 1.471 | 0.5 |  |
|  | Aβ_25-35_ + Stryphnodendron *adstringens* | TRG | 1 | 1.079 | 0.915 - 1.387 | 0.732 - 1.474 | 0.427 |  |
|  | 10 µM Aβ_25-35_ | TRG | 1 | 0.915 | 0.616 - 1.359 | 0.456 - 1.609 | 0.592 |  |
| *GSK3β* | Control | TRG | 1 | 0.875 | 0.582 - 1.259 | 0.455 - 1.587 | 0.43 |  |
|  | Aβ_25-35_ + Stryphnodendron *adstringens* | TRG | 1 | 0.866 | 0.587 - 1.233 | 0.484 - 1.410 | 0.39 |  |
|  | 10 µM Aβ_25-35_ | TRG | 0.807 | 1.039 | 0.848 - 1.318 | 0.604 - 1.551 | 0.766 |  |
| *APOE* | Control | TRG | 0.807 | 0.766 | 0.618 - 0.995 | 0.426 - 1.179 | 0.042 | DOWN |
|  | Aβ_25-35_ + Stryphnodendron *adstringens* | TRG | 0.807 | 1.188 | 0.920 - 1.467 | 0.641 - 1.828 | 0.159 |  |

**S1 Table. Data analysis of mRNA expression of Alzheimer’s disease-related genes using the standalone software REST 2009 with efficiency correction (continuation).**

| **Gene** |  | **Condition** | **Type** | **Reaction Efficiency** | **Expression** | **Std. Error** | **95% C.I.** | **p(H1)** | **Result (p < 0.05)** |
| --- | --- | --- | --- | --- | --- | --- | --- | --- | --- |
|  |  | 10 µM Aβ_25-35_ | TRG | 0.985 | 1.07 | 0.756 - 1.539 | 0.683 - 1.642 | 0.56 |  |
| *ACHE* |  | Control | TRG | 0.985 | 0.837 | 0.613 - 1.194 | 0.371 - 1.382 | 0.29 |  |
|  |  | Aβ_25-35_ + Stryphnodendron *adstringens* | TRG | 0.985 | 0.822 | 0.646 - 1.138 | 0.506 - 1.179 | 0.13 |  |
|  |  | 10 µM Aβ_25-35_ | TRG | 0.667 | 1.341 | 1.104 - 1.649 | 0.918 - 2.016 | 0.007 | UP |
| *A2M* |  | Control | TRG | 0.667 | 0.947 | 0.716 - 1.188 | 0.674 - 1.609 | 0.596 |  |
|  |  | Aβ_25-35_ + Stryphnodendron *adstringens* | TRG | 0.667 | 1.147 | 0.922 - 1.426 | 0.783 - 1.809 | 0.199 |  |

All expression levels, standard errors, 95% confidence intervals and p-values are described.
